# Two new rare-earth oxyborates Ba_4_BiTbO(BO_3_)_4_ and Ba_1.54_Sr_2.46_BiTbO(BO_3_)_4_ and luminescence properties of the Ba_4_BiTb_1−*x*_Eu_*x*_O(BO_3_)_4_ phosphors†

**DOI:** 10.1039/d3ra08265b

**Published:** 2024-02-19

**Authors:** Xuean Chen, Xuyang Yuan, Weiqiang Xiao, Xiaoyan Song

**Affiliations:** a Faculty of Materials and Manufacturing, Key Laboratory of Advanced Functional Materials, Ministry of Education of China, Beijing University of Technology 100124 Beijing China xueanchen@bjut.edu.cn; b Beijing Key Laboratory of Microstructure and Property of Solids, Beijing University of Technology 100124 Beijing China

## Abstract

Single crystals of two new terbium oxyborates Ba_4_BiTbO(BO_3_)_4_ and Ba_1.54_Sr_2.46_BiTbO(BO_3_)_4_ were obtained by the high-temperature solution method. They crystallize in the hexagonal *P*6_3_/*mmc* group (*Z* = 2) with lattice parameters of *a* = 5.41865(9) Å, *c* = 26.3535(5) Å, *V* = 670.12(3) Å^3^ and *a* = 5.36534(19) Å, *c* = 26.0661(10) Å, *V* = 649.83(5) Å^3^, respectively. Their crystal structures feature two kinds of layers: [Tb(BO_3_)_2_]_*n*_^3*n*−^ formed by corner-sharing TbO_6_ octahedra and BO_3_ triangles, as well as [Bi(BO_3_)_2_O]_*n*_^5*n*−^ consisting of Bi_2_O_13_ dimers and BO_3_ groups, with alkali-earth cations sitting inside and between the layers. In addition, solid solutions of Ba_4_BiTb_1−*x*_Eu_*x*_O(BO_3_)_4_ (0 ≤ *x* ≤ 0.2) were prepared *via* the solid-state reaction method. The obtained products were characterized by powder XRD, SEM, IR/Raman, XPS, DRS, and luminescence spectroscopy. It was found that as the Eu^3+^ doped content varies from *x* = 0 to 0.2, the emission color of the Ba_4_BiTb_1−*x*_Eu_*x*_O(BO_3_)_4_ phosphors can be adjusted from cyan to near-white and then to orange-red or from green to orange and then to red under the excitation of 349 and 377 nm, respectively. Furthermore, the emission intensities and chromaticity coordinates were found to be sensitive to the temperature for the phosphor Ba_4_BiTb_0.999_Eu_0.001_O(BO_3_)_4_ upon 377 nm excitation. The above results demonstrate that Ba_4_BiTb_1−*x*_Eu_*x*_O(BO_3_)_4_ phosphors have potential as multifunctional materials for solid-state lighting and temperature sensing applications.

## Abstract 2

Two new oxyborates Ba_4_BiTbO(BO_3_)_4_ and Ba_1.54_Sr_2.46_BiTbO(BO_3_)_4_ were synthesized and structurally characterized and luminescence properties of Ba_4_BiTb_1−*x*_Eu_*x*_O(BO_3_)_4_ (0 ≤ *x* ≤ 0.2) were investigated.

## Graphical abstract

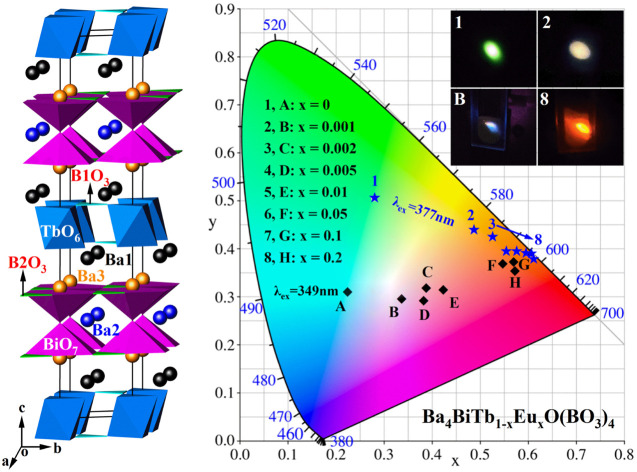

## 1. Introduction

Among solid-state lighting technology, white light-emitting diodes (w-LEDs) are excellent candidates to replace conventional incandescent lamps for their merits of high brightness, low energy consumption, long lifetime, small size, environmental-friendly nature, and so on.^1,2^ In the past years, cadmium chalcogenide quantum dots (QDs) have been proposed to be the promising alternatives to the phosphors in w-LEDs due to their tunable and narrow-band emission, high photoluminescence quantum yield (PLQY), and good stability.^3^ However, the cadmium chalcogenide QDs have some issues hindering their practical applications. For example, to achieve good stability and high PLQY, high reaction temperatures and delicate surface passivation (shelling) are often required. Lately, fully inorganic perovskite QDs, CsPbX_3_ (X = Cl, Br, I), have received rapidly growing attention as another option for further advancing w-LEDs.^4^ Different from chalcogenide QDs, perovskite QDs can be synthesized at much lower temperatures, and can easily achieve high PLQY without the need to grow passivation shells. Although different types of nanomaterials can be used for LEDs,^5–7^ to date, the most efficient and popular commercial w-LEDs are based on the combination of a blue-emitting InGaN or GaN chip with a yellow-emitting phosphor, *i.e.* Y_3_Al_5_O_12_:Ce^3+^ (YAG:Ce^3+^).^8^ However, the lack of red component in YAG-based w-LEDs results in a low color-rendering index (typically ≤80) and a high correlated color temperature (typically ≥4500 K), which in turn restricts their potential applicability in many fields, such as the general illumination or medical lighting.^9^ To solve this problem and obtain an ideal warm w-LEDs, an alternative strategy was proposed which is to employ red, green, and blue phosphors excited by ultraviolet (UV) or near-ultraviolet (n-UV) LED chips.^10^ This scheme is often prone to problems of complex mixing ratios and overlapping of energy levels among various phosphors.^11^ Besides, different attenuation situations of each phosphor will result in an unstable white light.^12^ Therefore, it has also been proposed to manufacture w-LEDs by coating single-phase white-light-emitting phosphors on UV/n-UV LED chips, which has always been a research hotspot in solid-state lighting.

Among the trivalent rare-earth ions, Tb^3+^ and Eu^3+^ are the most frequently used activators in luminescent materials that can produce green or red light under n-UV excitation due to the characteristic transitions of Tb^3+^:^5^D_4_ → ^7^F_*J*_ (*J* = 6, 5, 4, 3) or Eu^3+^:^5^D_0_ → ^7^F_*J*_ (*J* = 0, 1, 2, 3, 4).^13–16^ When Tb^3+^ and Eu^3+^ ions are co-doped into one host matrix, multi-color emission may occur owing to the efficient energy transfer from Tb^3+^ to Eu^3+^, the representative examples including KBaY(MoO_4_)_3_:Ln^3+^ (Ln^3+^ = Tb^3+^, Eu^3+^, Sm^3+^, Tb^3+^/Eu^3+^, Tb^3+^/Sm^3+^), Sr_3_Y(BO_3_)_3_:Tb^3+^, Eu^3+^, Sr_8_ZnY(PO_4_)_7_:Tb^3+^, Eu^3+^ and K_2_Tb_1−*x*_Eu_*x*_Hf(PO_4_)_3_.^17–20^ Once Tb^3+^ and Eu^3+^ ions are further combined with blue-emitting activators, such as Ce^3+^ or Eu^2+^, white light emission can be obtained, like the cases of BaY_2_Si_3_O_10_:Ce^3+^, Tb^3+^, Eu^3+^, MgY_4_Si_3_O_13_:Ce^3+^, Tb^3+^, Eu^3+^, Sr_1.5_Ca_0.5_SiO_4_:Eu^3+^, Tb^3+^, Eu^2+^ and Gd_2_O_2_S:Eu^3+^, Tb^3+^, Eu^2+^.^21–24^

The most common blue phosphors are usually produced by doping Ce^3+^ or Eu^2+^ into a host material.^25–27^ The syntheses of these phosphors require a reducing atmosphere, as the rare-earth ions Ce^3+^ and Eu^2+^ are in their lower valence states. As everyone knows, Bi^3+^ ion is a representative non-rare-earth activator with 6s^2^ electronic configuration, which can exhibit blue emission generated by the parity-allowed 6s^2^–6s6p inter-configurational transitions.^28^ Bi^3+^ has low toxicity and is stable in the air, and its synthesis condition is relatively mild compared to Ce^3+^ and Eu^2+^. Consequently, Bi^3+^ can also be used as a blue-emitting center to combine with Tb^3+^ and Eu^3+^ ions to obtain single-composition white phosphors, and some reported examples include β-GdB_3_O_6_:Bi^3+^, Tb^3+^, Eu^3+^, Ca_3_ZrSi_2_O_9_:Bi^3+^, Tb^3+^, Eu^3+^, Gd_2_MoB_2_O_9_:Bi^3+^, Tb^3+^, Eu^3+^ and NaYGeO_4_:Bi^3+^/Tb^3+^/Eu^3+^.^29–32^ However, doping with three ions such as Bi^3+^, Tb^3+^, and Eu^3+^ can lead to complicated energy transfer, so tuning to accurate white color remains still a challenge.

In the previous study, we systematically investigated the BaO–Bi_2_O_3_–PbO–Eu_2_O_3_–B_2_O_3_ system and discovered a new borate Ba_3_BiPbEuO(BO_3_)_4_.^33^ On this basis, we synthesized a series of Ba_3_BiPbY_1−*x*_Eu_*x*_O(BO_3_)_4_ (0 ≤ *x* ≤ 1) solid solutions and studied their luminescence properties. However, there have been no reports of doping Tb^3+^ activators in this host to obtain white or color-adjustable phosphors, and of course, temperature sensing properties of this type of phosphor have also not been investigated so far. Because Pb belongs to a toxic element that limits the practical application of this phosphor in w-LEDs. Our attempt to replace Pb^2+^ with iso-valent Ba^2+^ and Sr^2+^ ions led to the characterization of two new rare-earth oxyborates, Ba_4_BiTbO(BO_3_)_4_ and Ba_1.54_Sr_2.46_BiTbO(BO_3_)_4_. In this work, we first introduced the syntheses and crystal structures of these two new phases, and then, described the luminescence performance, energy transfer process, and temperature sensing behavior of Ba_4_BiTb_1−*x*_Eu_*x*_O(BO_3_)_4_ (0 ≤ *x* ≤ 0.2) fluorescent powders. It was found that the syntheses of this series of new borates can be carried out at a relatively low temperature without the need for a reducing atmosphere. Besides, the white light and color-tunable emission from green to red can be realized in Ba_4_BiTb_1−*x*_Eu_*x*_O(BO_3_)_4_ by simply changing the concentrations ratio of Tb^3+^/Eu^3+^ and selecting appropriate excitation irradiation. Additionally, a strong temperature sensitization phenomenon was found for this phosphor under 377 nm excitation, suggesting that Ba_4_BiTb_1−*x*_Eu_*x*_O(BO_3_)_4_ is a kind of luminescent material with potential application value in the field of w-LEDs and temperature sensors.

Note that Ba_4_BiTbO(BO_3_)_4_ and Ba_1.54_Sr_2.46_BiTbO(BO_3_)_4_ are the first quaternary (quinary) borate found in the BaO (and SrO)–Bi_2_O_3_–Tb_2_O_3_–B_2_O_3_ system, and also, the luminescence properties of Ba_4_BiTb_1−*x*_Eu_*x*_O(BO_3_)_4_ (0 ≤ *x* ≤ 0.2) are presented here for the first time. This study also reveals that Ba_4_BiLnO(BO_3_)_4_ (Ln = trivalent rare-earth ions) forms a large family of compounds, in which Ba^2+^ can be partially replaced by other divalent cations, such as Pb^2+^ and Sr^2+^. We believe that this work will enrich the structural chemistry of borates and help the development of new emission-tunable phosphors for w-LED applications and highly efficient luminescent thermometers for temperature-sensing applications.

## 2. Experimental section

### 2.1. Materials and methods

All chemicals for the synthesis were purchased from Sinopharm Chemical Reagent Co. Ltd, including BaCO_3_ (A.R.), SrCO_3_ (A.R.), Bi_2_O_3_ (A.R.), Y_2_O_3_ (99.99%), Tb_4_O_7_ (99.99%), Eu_2_O_3_ (99.99%) and H_3_BO_3_ (A.R.). Powder X-ray diffraction (XRD) measurements were conducted on a Bruker D8 ADVANCE diffractometer, which operated at 40 kV and 40 mA with Cu *K*_α_ radiation (*λ* = 1.5406 Å). Scanning electron microscope (SEM) images were taken using a Hitachi SU8020 instrument (accelerating voltage 20 kV) equipped with an energy-dispersive X-ray detector (EDX). Infrared (IR) spectra were obtained with a VERTEX 70 Fourier Transform Infrared Spectrometer (Bruker). Raman spectroscopy data were recorded on the powder sample by a Renishaw InVia Raman spectrometer, which has a spectral resolution of 1 cm^−1^ and is equipped with a confocal DM 2500 Leica optical microscope (with a 50× objective) and a 785 nm diode laser. Diffuse Reflectance Spectra (DRS) were measured with a Hitachi UH4150 UV-visible/NIR spectrophotometer, scanning at 120 nm min^−1^. The elemental valence states were analyzed *via* X-ray Photoelectron Spectroscopy (XPS) on a Thermo Scientific ESCALAB 250Xi spectrometer with monochromatized Al K_α_ radiation (*hν* = 1486.6 eV). Photoluminescence excitation (PLE) and emission (PL) spectra as well as luminescence decay curves were collected *via* the Edinburgh FLS 1000 fluorescence spectrometer. A 150 W Xe lamp and a 60 W μF flash lamp were used as the excitation source for the steady-state spectrum and decay curve measurements, respectively. Quantum yield (QY) and variable temperature PL spectra were measured by employing a barium sulfate-coated integrating sphere (150 mm in diameter) and a temperature controller (Oxford, OptistatDN2) attached to the FLS 1000 system, respectively.

### 2.2. Synthetic procedures

Single crystals of the title compounds were grown *via* the high-temperature solution method. Typically, a mixture of 0.2894 g BaCO_3_, 0.9112 g Bi_2_O_3_, 0.1828 g Tb_4_O_7_ and 0.0907 g H_3_BO_3_ (the molar ratio of 6 : 8 : 1 : 6) for Ba_4_BiTbO(BO_3_)_4_, and a mixture of 0.3879 g BaCO_3_, 0.2031 g SrCO_3_, 0.6411 g Bi_2_O_3_, 0.0735 g Tb_4_O_7_ and 0.1945 g H_3_BO_3_ (the molar ratio of 20 : 14 : 14 : 1 : 32) for Ba_1.54_Sr_2.46_BiTbO(BO_3_)_4_ were transferred to Pt crucibles after thorough grinding, respectively. In the furnace, these mixtures were gradually heated to 950 °C, where they were kept for 6 h, then cooled to 700 °C at a rate of 1.5 °C h^−1^, and further to 400 °C at 5 °C h^−1^, followed by cooling to room temperature at 20 °C h^−1^. The colorless transparent crystals of the title compounds were observed, which showed a hexagonal plate-like habit with various sizes ranging from 0.1 to 1.0 mm in diameter and several tenths of a millimeter in thickness. They were mechanically isolated from the solidified melt and further examined through SEM-EDX analyses, which confirmed the existence of heavy elements of Ba, Bi, Tb, and O (Ba, Sr, Bi, Tb, and O) (B is too light to be detected, see Fig. S1 and S2†).

Polycrystalline samples of Ba_4_BiYO(BO_3_)_4_ and Ba_4_BiTb_1−*x*_Eu_*x*_O(BO_3_)_4_ (*x* = 0, 0.001, 0.002, 0.005, 0.01, 0.05, 0.1, 0.2, and 1) were prepared by the traditional solid-state reaction method. The required metal carbonates or oxides and boric acid were weighted in stoichiometric ratio and thoroughly mixed and ground in an agate mortar for 30 min. Subsequently, they were transferred into alumina crucibles and preheated at 500 °C for 12 h to remove CO_2_ and H_2_O. Then further sintering at 830 °C for 96 h with multiple intermittent grinding was implemented to ensure the complete chemical reaction. Finally, the obtained products were naturally cooled to room temperature and ground again for further characterization.

### 2.3. Single-crystal X-ray diffraction

Single-crystal XRD data of two crystals were collected at room temperature on a SuperNova (Mo) X-ray source (*λ* = 0.71073 Å). Data reduction, cell refinements, and analytical absorption corrections were accomplished using CrysAlisPro software.^34^ The structures were solved by direct methods and refined with anisotropic displacement parameters for all atoms by full-matrix least-squares fitting on *F*^2^ using SHELX-2019.^35^

For Ba_4_BiTbO(BO_3_)_4_, the site-occupancy refinements indicated that there is an atomic site (Wyckoff 4e position) that was co-occupied by Ba and Bi atoms. In the initial refinement, Ba^2+^ and Bi^3+^ cations were placed at the same 4e site, with their atomic coordinates and anisotropic displacement parameters being constrained to be identical. After convergence of the refinement, rather large displacement parameters were obtained for this (Ba/Bi) site, suggesting additional positional disorder. Eventually, a significant improvement in the refinement was achieved after splitting the (Ba/Bi) into two close positions. A similar situation was found in Ba_1.54_Sr_2.46_BiTbO(BO_3_)_4_, except that (Ba/Bi) was replaced by (Sr/Bi). The structures were checked with the program MISSYM, and no obvious additional crystallographic symmetry was detected.^36^ General crystallographic information is presented in Table 1. Atomic coordinates, site occupancies, and equivalent isotropic displacement parameters, as well as selected bond lengths and angles, are provided in Tables S1–S3.†

**Table 1:** Crystallographic data for Ba_4_BiTbO(BO_3_)_4_ and Ba_1.54_Sr_2.46_BiTbO(BO_3_)_4_^a^

Formula	Ba_4_BiTbO(BO_3_)_4_	Ba_1.54(7)_Sr_2.46(7)_BiTbO(BO_3_)_4_
Formula weight	1168.50	1046.19
Crystal size (mm^3^)	0.25 × 0.20 × 0.05	0.25 × 0.25 × 0.15
Space group	*P*6_3_/*mmc* (no. 194)	*P*6_3_/*mmc* (no. 194)
*a* (Å)	5.41865(9)	5.36534(19)
*c* (Å)	26.3535(5)	26.0661(10)
*V* (Å^3^)	670.12(3)	649.83(5)
*Z*	2	2
*d*_calc_ (g cm^−3^)	5.791	5.347
*μ* (mm^−1^)	29.920	33.542
2*θ*_max_ (°)	70	66
Unique reflections	636	536
Observed [*I* ≥ 2*σ*(*I*)]	576	491
No. of variables	35	37
GOF on *F*_0_^2^	1.128	1.066
*R*_1_/w*R*_2_ [*I* ≥ 2*σ*(*I*)]	0.0417/0.0964	0.0604/0.1077
*R*_1_/w*R*_2_ (all data)	0.0464/0.0988	0.0645/0.1094

a *R*_1_ = Σ||*F*_0_| − |*F*_c_||/Σ|*F*_0_| and w*R*_2_ = [Σw(*F*_0_^2^ − *F*_c_^2^)^2^/Σw*F*_0_^4^]^1/2^ for *F*_0_^2^ > 2σ(*F*_0_^2^).

## 3. Results and discussion

### 3.1. Crystal structure description

**Fig. 1:**
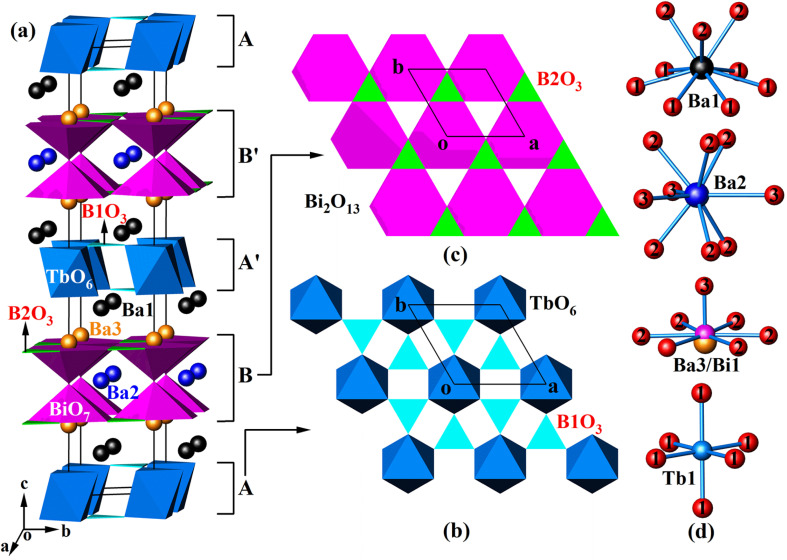
The unit cell of Ba_4_BiTbO(BO_3_)_4_ projected approximately along [100] (a), the sheets A and B viewed along [001] (b and c), and the local environment of each metal cation site (d). Ba1, Ba2, and Ba3: black, blue, and orange balls, respectively; BiO_7_: magenta hexagonal pyramids; TbO_6_: deep sky blue octahedra; B1O_3_ and B2O_3_: cyan and green triangles, respectively. In plot (c), when the structure is viewed along [001], two B2O_3_ triangles overlap, while two BiO_7_ hexagonal pyramids share a corner to form a Bi_2_O_13_ dimer.

Since Ba_4_BiTbO(BO_3_)_4_ and Ba_1.54_Sr_2.46_BiTbO(BO_3_)_4_ are isostructural, we chose Ba_4_BiTbO(BO_3_)_4_ to describe the crystal structure. The fundamental building blocks in this structure are TbO_6_ octahedra, BO_3_ triangles, and Bi_2_O_13_ dimers composed of two corner-sharing BiO_7_ hexagonal pyramids, as shown in Fig. 1(a). Within the *ab* plane, TbO_6_ octahedra are arranged hexagonally and further bridged by B1-centered BO_3_ groups *via* common O atoms to form a 2D infinite sheet of [Tb(BO_3_)_2_]_*n*_^3*n*−^ [layer A, see Fig. 1(b)]. There is another type of 2D sheet that has a composition [Bi(BO_3_)_2_O]_*n*_^5*n*−^ [layer B, see Fig. 1(c)]. This layer is constructed by corner-sharing Bi_2_O_13_ dimers, with a pair of boron atoms (two B2) accommodated within one-half of the three-membered rings of the layer. Four sheets, ABA′B′, are required to complete a translation period of the *c*-axis direction, where the sheets A′ and B′ are related to A and B by a 6_3_-screw axis. Some alkali-earth cations (Ba1 and Ba3) fill the space between the layers, while others (Ba2) reside in the channels that pass through the layers B and B′ to provide charge compensation.

As shown in Table S1,† there are nine unique atomic sites in the asymmetric unit of Ba_4_BiTbO(BO_3_)_4_, including 2Ba, 1(Ba/Bi), 1Tb, 2B, and 3O, all located in crystallographically special positions. Two distinct Ba atoms are both nine-coordinated, but adopt different coordination configurations: Ba1 in an irregular polyhedron and Ba2 in a tri-capped trigonal prism [Fig. 1(d)]. The Ba–O distances are normal, as shown in Table S2.† Concerning the disordered (Ba/Bi) site, it is found that Ba and Bi atoms are statistically distributed over two very close positions [Ba3–Bi1 = 0.320(3) Å]. Each is strongly bonded to one oxygen and also weakly bonded to six other O atoms to form a hexagonal pyramid, as shown in Fig. 1(d), which reflects the fact that the 6s^2^ non-bonded electron pairs of Bi^3+^ are stereochemically active. The Tb atom occupies the Wyckoff 2a position with local *D*_3d_ symmetry. It is surrounded by six oxygen atoms in an octahedral environment with six equal Tb–O bond distances of 2.255(6) Å, which are very close to those reported in K_3_TbB_6_O_12_ [2.229(4) Å–2.348(5)].^37^ Two B atoms are each connected to three oxygen atoms, forming a BO_3_ planar triangle with bond lengths in the range of 1.372(6)–1.379(7) Å, and the bond angles deviated slightly from 120°. These are common values as found in other known borates.^33,38^ The calculated total bond valences for Ba1, Ba2, Tb, B1, and B2 are 2.049, 2.052, 3.282, 2.991, and 2.937, respectively, consistent with their expected oxidation states.^39^

Although the title compounds are isostructural with our previously reported Pb^2+^-analog, Ba_3_BiPbEuO(BO_3_)_4_, their crystal structures show a certain difference.^33^ For example, in Ba_3_BiPbEuO(BO_3_)_4_, both Pb^2+^ and Bi^3+^ cations are located at the same 4e site with identical positional and thermal parameters, while in Ba_4_BiTbO(BO_3_)_4_ [Ba_1.54_Sr_2.46_BiTbO(BO_3_)_4_], this site is split into two very close positions, occupied by Ba^2+^ and Bi^3+^ (Sr^2+^ and Bi^3+^), respectively. This is understandable because both Pb^2+^ and Bi^3+^ have a 6s^2^ electron configuration and, of course, a similar coordination environment, while the ionic radii and coordination geometries around Ba^2+^ and Bi^3+^ as well as Sr^2+^ and Bi^3+^ are very different. Inspection of structural details (Tables S1–S3†) reveals that when Sr^2+^ partially replaces Ba^2+^ in Ba_4_BiTbO(BO_3_)_4_ to form Ba_1.54_Sr_2.46_BiTbO(BO_3_)_4_, the Ba1 and Ba2 sites are mixed with some Sr atoms, with the refined compositions Ba_0.58(2)_Sr_0.42(2)_ and Ba_0.38(3)_Sr_0.62(3)_, respectively. The Ba3/Bi1 site is still statistically distributed, but Ba3 is completely replaced by Sr3. Given identical coordinated numbers, (Ba/Sr)–O distances are significantly smaller than the corresponding Ba–O ones as expected. In addition, there is also a remarkable reduction in cell axis lengths and volume due to the smaller size of Sr^2+^ compared with Ba^2+^ (Table 1). What's more, this work also indicates that Ba_4_BiLnO(BO_3_)_4_ (Ln = trivalent rare-earth ions) forms a large class of compounds, where Ba^2+^ can be partially substituted by other divalent ions, like Pb^2+^ and Sr^2+^.

### 3.2. Phase purity and morphology

**Fig. 2:**
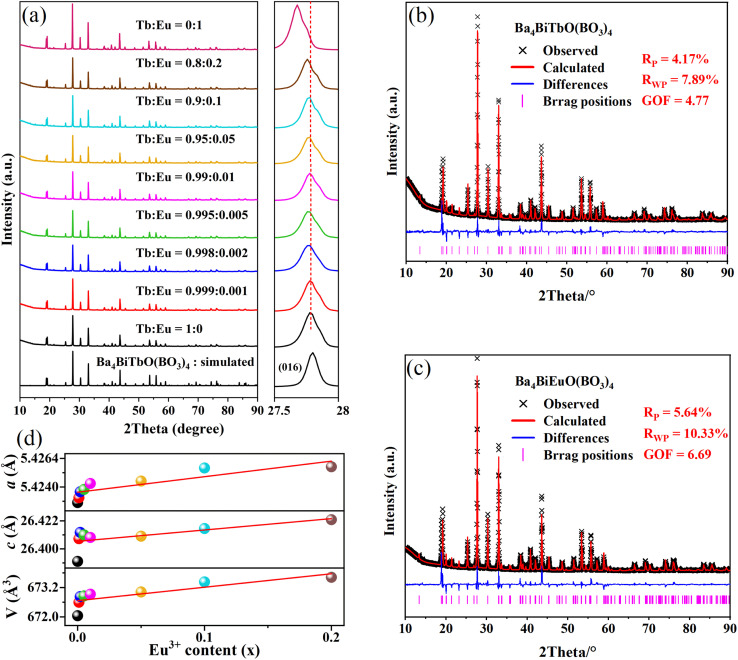
(a) XRD patterns of the Ba_4_BiTb_1−*x*_Eu_*x*_O(BO_3_)_4_ (0 ≤ *x* ≤ 1) phosphors. (b and c) Rietveld refinements of the XRD files for Ba_4_BiTb_1−*x*_Eu_*x*_O(BO_3_)_4_ (*x* = 0 and 1). (d) The cell parameters (*a*, *c*, and *V*) obtained from Rietveld fitting against the Eu^3+^ content (*x*).

A series of Ba_4_BiTb_1−*x*_Eu_*x*_O(BO_3_)_4_ (0 ≤ *x* ≤ 1) samples were prepared and their crystallinity and phase purity were assessed by powder XRD, as shown in Fig. 2(a). For comparison, the simulated pattern of Ba_4_BiTbO(BO_3_)_4_ based on its single-crystal structure is provided at the bottom of the figure. All observed XRD patterns are similar to each other and also consistent with the simulated pattern of the fully Tb^3+^ sample, showing that the introduction of Eu^3+^ into this host still maintains single-phase and does not cause significant changes in the crystal structure. Another noteworthy feature is that the enlarged diffraction peaks from 2*θ* = 27.5°–28.0° of the Ba_4_BiTb_1−*x*_Eu_*x*_O(BO_3_)_4_ samples, belonging to the representative (016) crystal face, slightly shift towards lower angles relative to the position of Ba_4_BiTbO(BO_3_)_4_ with increasing *x*. Since the effective ionic radius of Eu^3+^ (*r* = 0.947 Å, CN = 6) is larger than that of Tb^3+^ (*r* = 0.923 Å, CN = 6), but smaller than that of Bi^3+^ (*r* = 1.07 Å, CN = 7),^40,41^ such a shift implies that Eu^3+^ ions prefer to substitute Tb^3+^ instead of Bi^3+^ sites in the Ba_4_BiTbO(BO_3_)_4_ host. This is understandable because the coordination configuration around Eu^3+^ and Tb^3+^ is very similar and relatively regular, while that around Bi^3+^ is severely distorted due to the steric effect of 6s^2^ lone pair electrons [see Fig. 1(d)].

To further recognize the effect of doping Eu^3+^ ions on the crystal structures of Ba_4_BiTb_1−*x*_Eu_*x*_O(BO_3_)_4_, Rietveld refinements were conducted on the powder XRD profiles of Ba_4_BiTb_1−*x*_Eu_*x*_O(BO_3_)_4_ (*x* = 0, 0.001, 0.002, 0.005, 0.01, 0.05, 0.1, 0.2, and 1) using TOPAS Academic software.^42^ The initial model used for the Rietveld refinements was the single-crystal structure of Ba_4_BiTbO(BO_3_)_4_ (Tables 1 and S1†). The constraint condition is that atomic coordinates of B and O, all atomic occupancies, and isotropic displacement parameters were fixed, while the positional parameters of heavy atomic sites [including *z*(Ba1), *z*(Ba3), and *z*(Bi1)] and lattice constants (*a* and *c*) were refined along with other parameters. The final refinement results are presented in detail in Table S4† as well as Fig. 2(b), (c) and S3.† As representatives, the lattice parameters of the species for 0 ≤ *x* ≤ 0.2 are plotted against the substitution level *x* in Fig. 2(d). The refinements proceeded smoothly and the residual factors *R*_wp_ are all less than 10.4%, which further confirmed that this series of solid solutions are isomorphic and crystallize in the hexagonal system with space group *P*6_3_/*mmc*. In addition, the cell parameters (*a*, *c*, and *V*) show an obvious linear expansion along with the increase in Eu^3+^ concentration. These results conform to Vegard's rule, indicating that Eu^3+^ ions are completely dissolved in the Ba_4_BiTbO(BO_3_)_4_ host to form perfect solid solutions of Ba_4_BiTb_1−*x*_Eu_*x*_O(BO_3_)_4_.

**Fig. 3:**
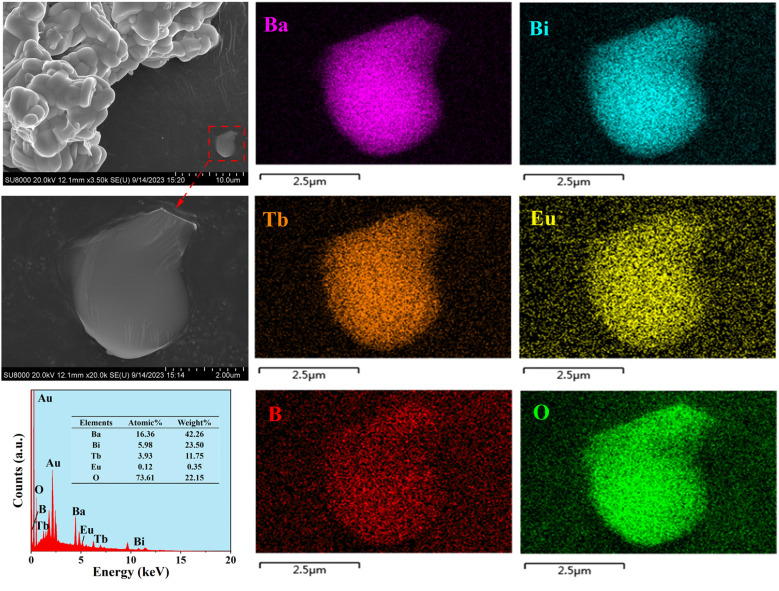
FE-SEM images, typical EDX results, and elemental mapping of the Ba_4_BiTb_0.999_Eu_0.001_O(BO_3_)_4_ phosphor. Note that a thin layer of Au was evaporated on the sample surface to provide conductivity before SEM inspection.

Because particle size and surface roughness are important factors influencing the luminescence performance of fluorescent powders, FE-SEM and EDX analyses were conducted to investigate the morphological characteristics of the obtained powder samples. Fig. 3 displays the FE-SEM images, EDX spectrum, and elemental mapping of the prepared Ba_4_BiTb_0.999_Eu_0.001_O(BO_3_)_4_ phosphor. It can be seen that the particles are aggregated together and have good crystallinity with smooth surface and irregular morphology, and the particle size is in the range of several micrometers. This may be due to the sample being obtained through a solid-state reaction route of intermediate grinding. The EDX pattern confirms the presence of the dominant elements Ba, Bi, Tb, Eu, B, and O, all homogeneously distributed over the same selection region of the sample, as demonstrated in the elemental mapping images. This indicates that the target solid solution has been successfully synthesized (note that the boron content cannot be accurately measured due to its low atomic weight, while the gold signal comes from the gold sputtering process).

### 3.3. IR and Raman spectra

Although single crystal XRD analyses indicated the presence of BO_3_ groups in the title compounds, IR and Raman spectroscopy were still studied to further confirm the coordination environment of B atoms. Ba_4_BiLnO(BO_3_)_4_ (Ln = trivalent rare-earth ions) crystallizes in the centrosymmetric *P*6_3_/*mmc* (*D*_6h_^4^, No. 194) group (*Z* = 2). Based on group theoretical analysis,^33,43^ 138 Brillouin zone center modes are predicted, which have the mechanical representation: *Γ*_vibr_ = 8A_1g_ + 2A_1u_ + 2A_2g_ + 11A_2u_ + 10B_1g_ + 2B_1u_ + 2B_2g_ + 9B_2u_ + 11E_2u_ + 12E_2g_ + 13E_1u_ + 10E_1g_, where the E mode is doubly degenerate and usually observed as one frequency. These modes can be further subdivided into 2E_2u_ + 2E_2g_ + 2E_1u_ + 2E_1g_ asymmetric stretching (*ν*_3_), 2A_1g_ + 2A_2u_ + 2B_1g_ + 2B_2u_ symmetric stretching (*ν*_1_), 2A_1g_ + 2A_2u_ + 2B_1g_ + 2B_2u_ out-of-plane bending (*ν*_2_), 2E_2u_ + 2E_2g_ + 2E_1u_ + 2E_1g_ in-plane bending (*ν*_4_), 2A_1g_ + 2A_2u_ + 2B_1g_ + 2B_2u_ + 2E_2u_ + 2E_2g_ + 2E_1u_ + 2E_1g_ translational and 2A_1u_ + 2A_2g_ + 2B_1u_ + 2B_2g_ + 2E_2u_ + 2E_2g_ + 2E_1u_ + 2E_1g_ librational modes of the BO_3_ groups. The remaining modes correspond to translations of the Ba1 (A_1g_ + A_2u_ + B_1g_ + B_2u_ + E_2u_ + E_2g_ + E_1u_ + E_1g_), Ba2 (A_2u_ + B_1g_ + E_2g_ + E_1u_), (Ba3/Bi1) (A_1g_ + A_2u_ + B_1g_ + B_2u_ + E_2u_ + E_2g_ + E_1u_ + E_1g_), Tb1 (A_2u_ + B_2u_ + E_2u_ + E_1u_) and O3 (A_2u_ + B_1g_ + E_2g_ + E_1u_) sites. It should be remembered that three vibrational modes (A_2u_ + E_1u_) belong to the acoustic branch, therefore the total number of optical modes in the crystal is 135 (*Γ*_optic_ = 8A_1g_ + 2A_1u_ + 2A_2g_ + 10A_2u_ + 10B_1g_ + 2B_1u_ + 2B_2g_ + 9B_2u_ + 11E_2u_ + 12E_2g_ + 12E_1u_ + 10 E_1g_). Since A_1u_, A_2g_, B_1g_, B_1u_, B_2g_, B_2u,_ and E_2u_ modes are silent, there are a total of 86 optical modes distributed into 52 Raman-active (*Γ*_R_ = 8A_1g_ + 12E_2g_ + 10E_1g_) and 34 IR-active (*Γ*_IR_ = 10A_2u_ + 12E_1u_) modes, leading to 30 Raman and 22 IR frequencies, respectively. It is difficult to accurately assign the individual bands to specific vibrational modes, as some modes have very similar wavenumbers, but a rough assignment of groups for both IR and Raman spectra is possible.

Fig. S4† presents the polycrystalline IR and Raman spectra measured in the internal mode region for Ba_4_BiLnO(BO_3_)_4_ (Ln = Y, Tb and Eu). Three compounds show similar spectral profiles, illustrating that they are isostructural to each other, and the introduction of different rare-earth ions does not affect the fundamental vibrational modes of the main structural units. Both IR and Raman spectra above 550 cm^−1^ can be divided into four regions, of which the area of 1100–1400 cm^−1^ shows the asymmetric stretching vibrations (*ν*_3_) of the BO_3_ groups. The absorption bands between 850 and 980 cm^−1^ can be assigned as the BO_3_ symmetric stretching vibrations (*ν*_1_). The absorption caused by the out-of-plane bending (*ν*_2_) of the trigonal group appears as bands in the range of 700–800 cm^−1^. The remaining bands from 550 to 680 cm^−1^ are the result of the in-plane BO_3_ bending (*ν*_4_). These assignments are consistent with those of other reported borates.^44–46^ As everyone knows, for the ideal BO_3_ group with *D*_3h_ symmetry, the modes *ν*_1_(A_1_′) and *ν*_2_(A_2_′′) should be IR and Raman inactive, respectively.^43^ The appearance of vibrations *ν*_1_(2A_2u_) in the IR and *ν*_2_(2A_1g_) in the Raman spectra indicates that the BO_3_ groups in Ba_4_BiLnO(BO_3_)_4_ deviate from the ideal symmetry. In fact, our structural analysis reveals that there are two crystallographically independent B atoms, both of which occupy the 4f position with local symmetry *C*_3v_ instead of *D*_3h_ (see Table S1†).

### 3.4. XPS spectra

To get an idea of the valence states of the constituent elements in the system, we conducted XPS studies on the phosphor Ba_4_BiTb_0.999_Eu_0.001_O(BO_3_)_4_ over a wide energy range, as shown in Fig. S5(a).† The XPS survey scan shows the respective photoemission peaks caused by the constituent elements (Ba, Bi, Tb, Eu, B, and O) and the Auger peaks of Ba and O (Ba MNN & O KLL). The appearance of the C 1s signal is due to the absorption of adventitious hydrocarbons on the sample surface during the sample handling for XPS measurements. The chemical composition and bonding information were further probed utilizing the detailed, high-resolution core-level XPS scans of Ba 3d, Bi 4f, Tb 3d, Eu 3d, B 1s, and O 1s, as illustrated in Fig. S5(b)–(g).† The Ba 3d can be deconvoluted into two spin-orbital splitting components, Ba 3d_3/2_ and Ba 3d_5/2_, located at the binding energies (BEs) of 795.48 and 780.25 eV, respectively.^47^ The XPS spectrum related to Bi 4f consists of two intense and symmetrical peaks observed at 164.47 (Bi 4f_5/2_) and 159.25 (Bi 4f_7/2_) eV with an energy difference of ∼5.22 eV.^48^ The Tb 3d XPS peaks also exhibit doublet characteristics, which are assigned to Tb 3d_3/2_ (∼1278.86 eV) and Tb 3d_5/2_ (∼1244.18 eV), separated by 34.68 eV.^49^ These BE values can be used as fingerprints to identify the presence of Ba^2+^, Bi^3+^, and Tb^3+^, respectively. In Fig. S5(e),† due to the very low Eu^3+^ content in the synthesized sample, the Eu 3d XPS signal is relatively weak, but it can be resolved into two components, corresponding to Eu 3d_3/2_ (∼1168.40 eV) and 3d_5/2_ (∼1138.02 eV), similar to previous results of other Eu^3+^-based materials.^33,50^ For the B 1s and O 1s spectra, they can be fitted to symmetric single peaks with BEs of 191.32 and 531.60 eV, respectively, which are typical values related to B–O or O–cation bonding states in borates.^51–53^ In addition, no other impurity peaks (including those from Eu^2+^ and Tb^4+^ ions) were identified from the XPS spectra. These results in combination with XRD and EDX data ensure the successful preparation of Ba_4_BiTb_1−*x*_Eu_*x*_O(BO_3_)_4_ phosphors, which provides a solid basis for subsequent investigations of optical absorption and PL properties.

### 3.5. UV-vis diffuse reflection spectra

To investigate the optical absorption properties, the UV-vis diffuse reflection spectra of Ba_4_BiLnO(BO_3_)_4_ (Ln = Y, Tb, and Eu) were measured, as shown in Fig. S6.† All samples show a very high reflectance between 90 and 100% relative to the white standard BaSO_4_ (optical grade) in the visible region, indicating a high optical grade of the as-prepared compounds. Below 400 nm, there is an intense broad absorption band, which is due to the collective effect of the ^1^S_0_ → ^1^P_1_/^3^P_1_ transition of Bi^3+^ and the host lattice absorption.^28^ For the Tb and Eu compounds, the Tb^3+^:4f^8^ → 4f^7^5d^1^ absorption and O^2−^ → Eu^3+^ charge transfer absorption are also located in this area, respectively. Moreover, several sharp characteristic absorption peaks at about 393, 466, 527, 535 and 592 nm are assigned to ^7^F_0_ → ^5^L_6_, ^7^F_0_ → ^5^D_2_, ^7^F_0_ → ^5^D_1_, ^7^F_1_ → ^5^D_1_ and ^7^F_1_ → ^5^D_0_ transitions of Eu^3+^, respectively, while the peak at 486 nm is ascribed to the ^7^F_6_ → ^5^D_4_ transition of Tb^3+^.^54^

The optical band gap of Ba_4_BiLnO(BO_3_)_4_ can be estimated by the following formula:^55^

1 [*F*(*R*_∞_)*hν*]*n* = *A*(*hν* − *E*_g_)

where *hν* is the photon energy, *A* is a proportional constant, *E*_g_ is the optical band gap energy and the value of *n* is 1/2 or 2 for an indirect or a direct allowed transition, respectively. The *F*(*R*_∞_) can be described by the Kubelka–Munk function:^56^

2 *F*(*R*_∞_) = (1 − *R*)2/2*R* = *K*/*S*

here, *R*, *K*, and *S* are the reflection, absorption, and scattering coefficients, respectively. From the linear extrapolation of the [*F*(*R*_∞_)*hν*]^*n*^–*hν* plot to [*F*(*R*_∞_)*hν*]^*n*^ = 0, the indirect and direct band gaps of Ba_4_BiYO(BO_3_)_4_ are estimated to be approximately 3.36 and 3.55 eV, while they are 3.34 (3.39) and 3.53 eV (3.59) for its Tb (Eu)-analog, respectively (see Fig. S6†). These *E*_g_ values are comparable to those previously reported in Ba_3_BiPbEuO(BO_3_)_4_ [3.02 (indirect) and 3.35 (direct) eV].^33^

### 3.6. PLE and PL spectra

As is known to all, Bi^3+^ possesses 6s^2^ lone pair electrons, therefore, the position of its excitation and emission bands depends largely on the nature of the host lattice. The ground state of Bi^3+^ is ^1^S_0_ and its excited states are composed of ^3^P_0_, ^3^P_1_, ^3^P_2_, and ^1^P_1_ in order of increasing energy. For the electron transition of Bi^3+^, ^1^S_0_ → ^1^P_1_ is parity- and spin-allowed, but it is usually located in the deep ultraviolet region (typically ≤250 nm), so it is difficult to use in LED devices. ^1^S_0_ → ^3^P_2_ is spin-forbidden and the luminous intensity is low. ^1^S_0_ → ^3^P_0_ is strictly forbidden because the total angular momentum does not change (Δ*J* = 0). In contrast, ^1^S_0_ → ^3^P_1_ has the longest luminous wavelength and can even enter the n-UV region (around 350–420 nm). Although it is forbidden according to the spin-selection rule, it will become partially allowed by mixing with singlet and triplet states (Δ*J* = 1).^57^

**Fig. 4:**
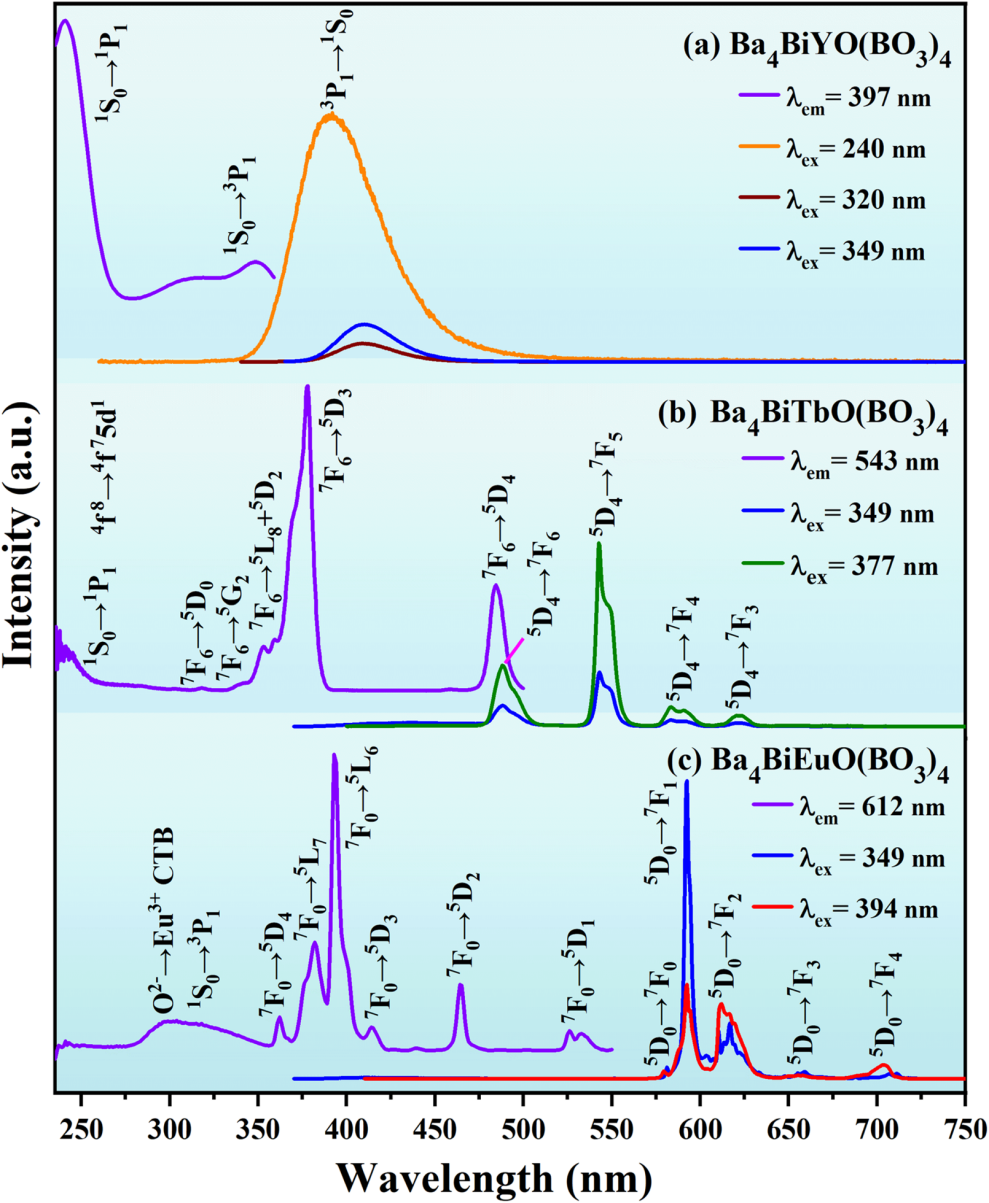
The PLE and PL spectra of Ba_4_BiYO(BO_3_)_4_ (a), Ba_4_BiTbO(BO_3_)_4_ (b) and Ba_4_BiEuO(BO_3_)_4_ (c).

The luminescent properties of the title compounds were characterized by the photoluminescence excitation (PLE) and emission (PL) spectra. As depicted in Fig. 4(a), the PLE spectrum of Ba_4_BiYO(BO_3_)_4_ (*λ*_em_ = 397 nm) shows three broad excitation bands, attributing to the ^1^S_0_ → ^1^P_1_ transition of Bi^3+^ (240 nm), and ^1^S_0_ → ^3^P_1_ transitions of Bi^3+^ (I) (320 nm) and Bi^3+^ (II) (349 nm), respectively,^58–61^ which are related to the crystal structure where the disordered (Ba/Bi) site is split into two positions (Table S1†). Upon 240 nm excitation, the PL spectrum presents an asymmetric emission band extending from 340 to 530 nm with the maximum at about 397 nm, which is ascribed to the ^3^P_1_ → ^1^S_0_ transition of Bi^3+^.^58–61^ The profiles of the emission spectra under 320 and 349 nm excitation are similar to those under 240 nm excitation, except that the emission intensities are much lower.

Fig. 4(b) shows the luminescence spectra of Ba_4_BiTbO(BO_3_)_4_. The PLE spectrum monitored at 543 nm comprises two parts: the absorption band from 230 to 275 nm (centered at ∼236 nm) corresponding to the 4f^8^ → 4f^7^5d^1^ transition of Tb^3+^ overlapped with the ^1^S_0_ → ^1^P_1_ transition of Bi^3+^, and the other from 275 to 500 nm mainly associated with the intra-configurational 4f^8^ → 4f^8^ transitions of Tb^3+^ from ^7^F_6_ levels to ^5^D_0_ (318 nm), ^5^G_2_ (342 nm), ^5^L_8_ (353 nm), ^5^D_2_ (360 nm), ^5^D_3_ (377 nm) and ^5^D_4_ (485 nm) levels.^54,62^ The PL spectrum excited at 377 nm exhibits four groups of emission peaks in the region from 450 to 650 nm, of which the strongest at 543 nm is due to the ^5^D_4_ → ^7^F_5_ transition, while others at 488, 584, and 621 nm belong to the ^5^D_4_ → ^7^F_6_, ^5^D_4_ → ^7^F_4_, and ^5^D_4_ → ^7^F_3_ transitions, respectively.^54,62^ The spectrum clearly shows strong ^5^D_4_ emission, and no significant ^5^D_3_ emission was observed due to efficient cross-relaxation processes [^5^D_3_(Tb^3+^) + ^7^F_6_(Tb^3+^) → ^5^D_4_(Tb^3+^) + ^7^F_0_(Tb^3+^)] as expected for a fully concentrated Tb^3+^ material.^63^ In addition, the PL spectrum under 349 nm excitation (Bi^3+^ absorption) includes intense Tb^3+^ emission together with weak Bi^3+^ emission, suggesting the occurrence of Bi^3+^ to Tb^3+^ energy transfer in Ba_4_BiTbO(BO_3_)_4_.

Fig. 4(c) presents the luminescence spectra of Ba_4_BiEuO(BO_3_)_4_. When monitoring 612 nm emission, the PLE spectrum consists of one broad absorption band in the region of 280–357 nm (centered at ∼299 nm) and several sharp excitation peaks in the longer wavelength region. The wide band is the typical absorption of O^2−^ → Eu^3+^ charge transfer superimposed with the ^1^S_0_ → ^3^P_1_ transition of Bi^3+^, while the sharp peaks at 362, 382, 394, 414, 465, and 526 nm are attributed to ^7^F_0_ → ^5^D_4_, ^5^L_7_, ^5^L_6_, ^5^D_3_, ^5^D_2_ and ^5^D_1_ transitions of Eu^3+^, respectively.^54,62^ Upon 394 nm excitation, the PL spectrum shows a series of sharp emission peaks within 570–710 nm. These peaks located at 580, 593, 612, 656, and 704 nm are readily assigned to ^5^D_0_ → ^7^F_*J*_ (*J* = 0–4) transitions of Eu^3+^, respectively. When excited at 349 nm (Bi^3+^ absorption), besides the very small residual emission of Bi^3+^, the strong characteristic emission peaks of Eu^3+^ can also be observed, which means an efficient energy transfer from Bi^3+^ to Eu^3+^. The comparison of the PLE spectrum for Ba_4_BiLnO(BO_3_)_4_ (Ln = Tb and Eu) and PL spectrum for Ba_4_BiYO(BO_3_)_4_ reveals a significant spectral overlap between the emission band of Bi^3+^ and the f–f absorption of Tb^3+^ and Eu^3+^ in the wavelength range of 360–425 nm. Therefore, the effective energy transfer from Bi^3+^ to both Tb^3+^ and Eu^3+^ can be expected. It is noticeable that among the observed emission transitions, the magnetic dipole ^5^D_0_ → ^7^F_1_ is predominant, suggesting that the surrounding environment of Eu^3+^ is centrosymmetric. This is in line with our crystallographic research, which shows that Eu^3+^ may replace Tb^3+^ and mainly occupies the six-fold coordinated Wyckoff 2a site, which has a regular octahedral coordination configuration. Therefore, the ligand field around Eu^3+^ is centrosymmetric [see Fig. 1(d) and Table S1†].

**Fig. 5:**
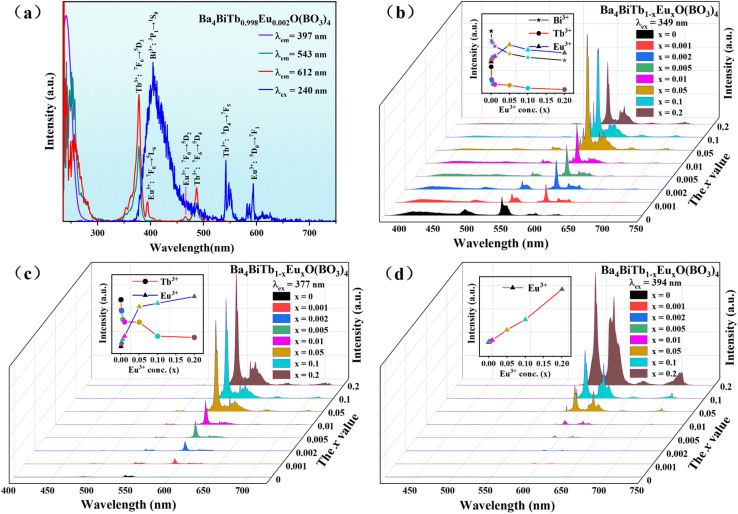
The PLE and PL spectra of the Ba_4_BiTb_0.998_Eu_0.002_O(BO_3_)_4_ phosphor (a) as well as the concentration-dependent PL spectra of the Ba_4_BiTb_1−*x*_Eu_*x*_O(BO_3_)_4_ (0 ≤ *x* ≤ 0.2) fluorescent powders [(b) *λ*_ex_ = 349 nm; (c) *λ*_ex_ = 377 nm; (d) *λ*_ex_ = 394 nm]. The integrated emission intensities of Bi^3+^ in the range of 370–475 nm, Tb^3+^ at about 543 nm (^5^D_4_ → ^7^F_5_), and Eu^3+^ at about 593 nm (^5^D_0_ → ^7^F_1_) were also calculated and shown in the insets of the figure.

Fig. 5(a) shows the PLE and PL spectra of the representative phosphor, Ba_4_BiTb_0.998_Eu_0.002_O(BO_3_)_4_. The PLE spectra monitored with Bi^3+^ emission of 397 nm and Tb^3+^ emission of 543 nm are similar to those of Ba_4_BiYO(BO_3_)_4_ and Ba_4_BiTbO(BO_3_)_4_, respectively, which means that the energy transfer of Bi^3+^ → Tb^3+^ is still apparent in this Bi^3+^/Tb^3+^/Eu^3+^ coexisting phosphor. On the contrary, the PLE spectrum of Ba_4_BiTb_0.998_Eu_0.002_O(BO_3_)_4_ by monitoring Eu^3+^ emission at 612 nm differs slightly from those of Ba_4_BiTbO(BO_3_)_4_ and Ba_4_BiEuO(BO_3_)_4_. It is primarily composed of the characteristic excitation bands of Tb^3+^:4f^8^ → 4f^7^5d^1^ and 4f^8^ → 4f^8^ transitions, with small admixtures of those of Bi^3+^:^1^S_0_ → ^1^P_1_ and Eu^3+^:4f^6^ → 4f^6^ transitions. On the other hand, under 240 nm (Bi^3+^:^1^S_0_ → ^1^P_1_) excitation, the PL spectrum of Ba_4_BiTb_0.998_Eu_0.002_O(BO_3_)_4_ contains not only the Bi^3+^ blue emission band at 370–475 nm but also the Tb^3+^ green emission peak at 543 nm and the Eu^3+^ orange emission peak at 593 nm. The presence of Bi^3+^ and Tb^3+^ absorption bands when monitoring the Eu^3+^ emission and the observation of both Tb^3+^ and Eu^3+^ characteristic emissions when excited by Bi^3+^ absorption suggests the occurrence of Bi^3+^ → Eu^3+^, Tb^3+^ → Eu^3+^ and Bi^3+^ → Tb^3+^ energy transfer in the three-ion coexisting phosphor.

Since the UV-excitation at ∼240 nm does not match the output wavelength of commercial n-UV (*λ*_em_ = 350–420 nm) LED chips, and also the Ba_4_BiTb_1−*x*_Eu_*x*_O(BO_3_)_4_ (0 ≤ *x* ≤ 0.2) phosphors show a blue-to-purple emission (without passing through the white light region) under this excitation, we chose 349 nm (Bi^3+^:^1^S_0_ → ^3^P_1_) as the excitation wavelength for Bi^3+^ to measure the concentration-dependent PL spectra of Ba_4_BiTb_1−*x*_Eu_*x*_O(BO_3_)_4_. As seen from Fig. 5(b), the spectra exhibit typical Bi^3+^:^3^P_1_ → ^1^S_0_, Tb^3+^:^5^D_4_ → ^7^F_*J*_ (*J* = 6, 5) and Eu^3+^:^5^D_0_ → ^7^F_*J*_ (*J* = 1–4) transitions in the blue, green, and orange emission regions, respectively. With an increase in the Eu^3+^ content (*x*), the emission intensities of both Bi^3+^ and Tb^3+^ decline monotonously, whereas that of Eu^3+^ first increases until *x* = 0.05 and then appears a downfall. This is easy to understand because the occurrence of Bi^3+^ → Tb^3+^ and Bi^2+^ → Eu^3+^ energy transfer results in a reduction of Bi^3+^ emissions. The incorporation of Eu^3+^ into the Ba_4_BiTbO(BO_3_)_4_ host would cause the substantial energy transfer from Tb^3+^ to Eu^3+^, and meanwhile, the relatively high efficiency of Bi^3+^-to-Eu^3+^ energy transfer would suppress the Bi^3+^-to-Tb^3+^ energy transfer, therefore, a decrease in Tb^3+^ emissions can be anticipated. In addition, the increase of Eu^3+^ emissions with increasing Eu^3+^ content up to *x* = 0.05 is also understandable because both Bi^3+^ and Tb^3+^ can transfer energy to Eu^3+^. However, once the Eu^3+^ content (*x*) exceeds 0.05, the concentration quenching occurs, resulting in the weakening of the Eu^3+^ emission intensity. Moreover, as shown in Fig. 5(c), when excited at 377 nm (Tb^3+^:^7^F_6_ → ^5^D_3_), the PL spectra exhibit both Tb^3+^ and Eu^3+^ emissions, with the latter dominating. As the Eu^3+^ concentration increases, the emission intensity of Tb^3+^ at 543 nm decreases, while that of Eu^3+^ at 593 nm shows the opposite trend, as a consequence of Tb^3+^ → Eu^3+^ energy transfer. However, if we use 394 nm (Eu^3+^:^7^F_0_ → ^5^L_6_) as the excitation wavelength, only the characteristic emissions of Eu^3+^:^5^D_0_ → ^7^F_*J*_ (*J* = 0–4) are visible in Fig. 5(d), indicating that the inverse transition (Eu^3+^ → Bi^3+^ and Eu^3+^ → Tb^3+^) is forbidden. In this case, the PL intensity increases linearly with the Eu^3+^ doping content (*x*), and no concentration quenching occurs up to *x* = 0.2. These observations show that the emission profiles and color of Ba_4_BiTb_1−*x*_Eu_*x*_O(BO_3_)_4_ are influenced not only by the dopant concentration but also by the selected excitation wavelength. Similar phenomena were reported in CdTb_1−*x*_Sm_*x*_GaB_2_O_7_, CaCO_3_:Eu^3+^, Tb^3+^ and CeO_2_:Eu^3+^ as well.^64–66^

### 3.7. Fluorescence lifetime and energy transfer mechanism

**Fig. 6:**
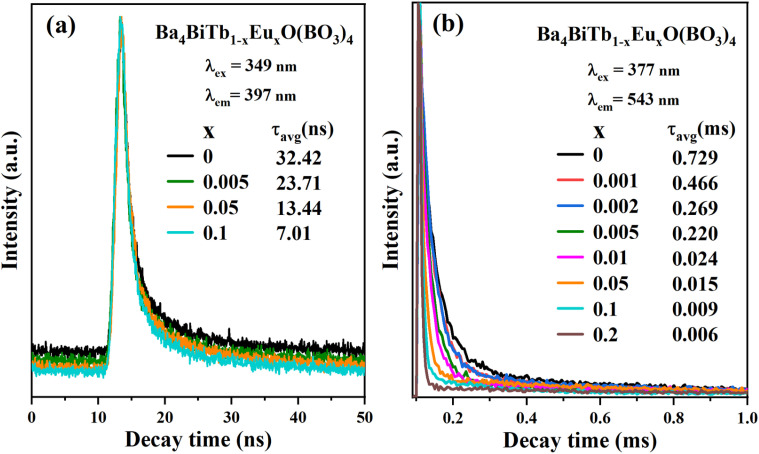
Decay curves of the Ba_4_BiTb_1−*x*_Eu_*x*_O(BO_3_)_4_ phosphors monitoring ^3^P_1_ → ^1^S_0_ transition of Bi^3+^ (a) and ^5^D_4_ → ^7^F_5_ transition of Tb^3+^ (b) under excitation at 349 and 377 nm, respectively.

To further illustrate the energy transfer processes, Fig. 6 shows the fluorescence decay curves for the Bi^3+^:^3^P_1_ → ^1^S_0_ and Tb^3+^:^5^D_4_ → ^7^F_5_ emission bands of Ba_4_BiTb_1−*x*_Eu_*x*_O(BO_3_)_4_ under excitation at 349 and 377 nm, respectively. All the decay profiles can be fitted into a triple-exponential function:3
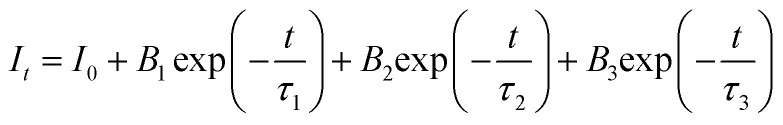
in this expression, *I*_0_ and *I*_*t*_ represent the PL intensity at time 0 and *t*, respectively. *B*_1_, *B*_2_, and *B*_3_ stand for the fitting constants. *τ*_1_, *τ*_2_, and *τ*_3_ are lifetimes for the triple-exponential components. The average decay lifetime (*τ*_avg_) can be calculated using the equation given below:^67^4
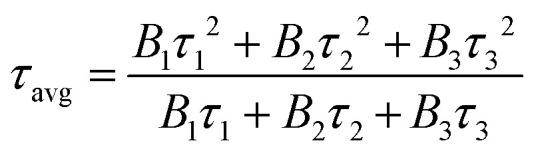


The *τ*_avg_ values obtained for different phosphors are also provided in Fig. 6, from which it is easy to see that increasing the Eu^3+^ concentration causes a reduction in the decay lifetimes of Bi^3+^ and Tb^3+^ emissions, which strongly supports the energy transfer from Bi^3+^ to Tb^3+^/Eu^3+^ and from Tb^3+^ to Eu^3+^.

**Fig. 7:**
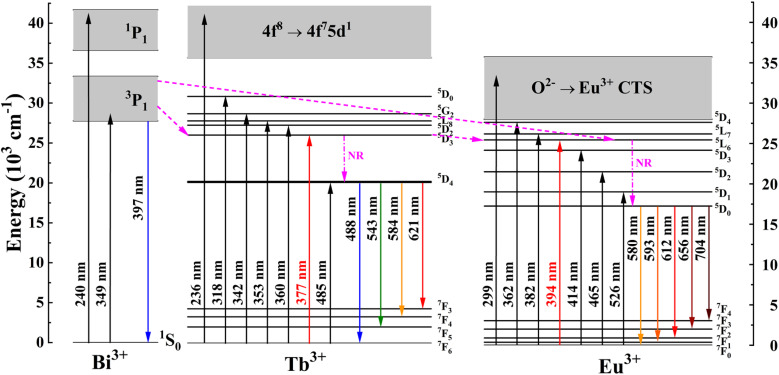
Schematic energy-level diagrams of Bi^3+^, Tb^3+^, and Eu^3+^ in Ba_4_BiTb_1−*x*_Eu_*x*_O(BO_3_)_4_, showing energy-transfer process (NR: nonradiative).

Based on the above analyses, a schematic representation of the possible energy transfer pathways in Ba_4_BiTb_1−*x*_Eu_*x*_O(BO_3_)_4_ is described in Fig. 7. Upon excitation at 349 nm, the electrons on Bi^3+^ ions can be effectively excited from the ground state ^1^S_0_ to the excited state ^3^P_1_. Then they relax nonradiatively to the lowest vibration mode of the ^3^P_1_ state. Afterward, some of the excited electrons return to the ground state of Bi^3+^ to generate the broadband blue emission, while the others can transfer their energy to the ^5^D_3_ level of Tb^3+^ and the ^5^L_6_ level of Eu^3+^ due to energy level matching, followed by non-radiative relaxation from ^5^D_3_ to ^5^D_4_ (Tb^3+^) and from ^5^L_6_ to ^5^D_0_ (Eu^3+^) states. Finally, a series of characteristic emissions of Tb^3+^:^5^D_4_ → ^7^F_6,5,4,3_ and Eu^3+^:^5^D_0_ → ^7^F_0,1,2,3,4_ occur. Apart from the energy transfer from Bi^3+^ to both Tb^3+^ and Eu^3+^, some of the excited electrons can also transfer their energy from the ^5^D_3_ level of Tb^3+^ to the ^5^L_6_ excited level of Eu^3+^, which enhances the Eu^3+^ emission, accompanied by a decrease in the Bi^3+^ and Tb^3+^ emissions.

### 3.8. Emitting light color analysis

In general, the Commission Internationale de I'Eclairage (CIE) chromaticity coordinates are used to characterize the emission color of the phosphor material, and the Correlated Color Temperature (CCT) is used to study which type of light the phosphor can emit. The CIE coordinates of the Ba_4_BiTb_1−*x*_Eu_*x*_O(BO_3_)_4_ (0 ≤ *x* ≤ 0.2) phosphors were determined by their corresponding PL spectra, and the CCT values were calculated using McCamy's empirical formula:^68^

**Fig. 8:**
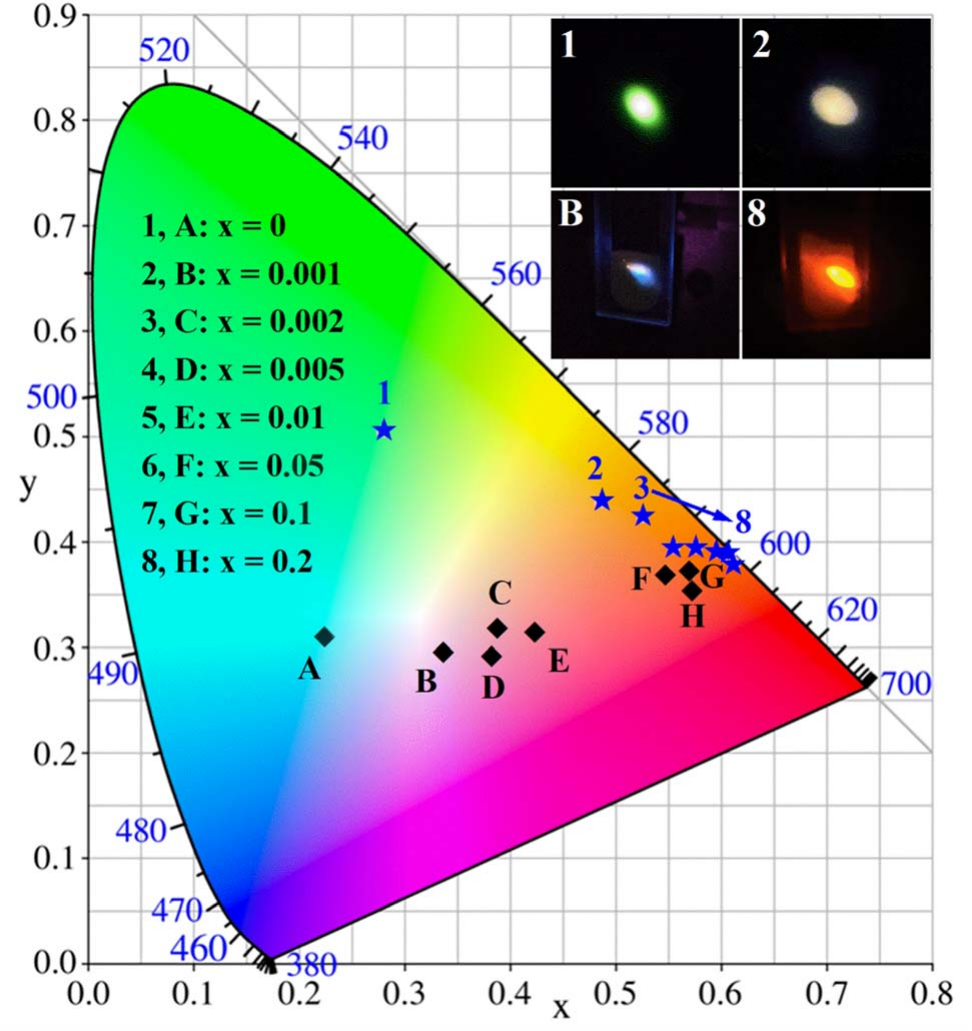
The CIE chromaticity diagrams of Ba_4_BiTb_1−*x*_Eu_*x*_O(BO_3_)_4_ (*x* = 0, 0.001, 0.002, 0.005, 0.01, 0.05, 0.1 and 0.2) phosphors (*λ*_ex_ = 349 nm for points A–H; *λ*_ex_ = 377 nm for points 1–8). The inset shows the photographs of the selected phosphors under the irradiation of 349 nm (point B) and 377 nm (points 1, 2, and 8), respectively.

5 CCT = −449*n*^3^ + 3525*n*^2^ − 6823.3*n* + 5520.33

where *n* = (*x* − *x*_e_)/(*y* − *y*_e_) and (*x*_e_ = 0.332, *y*_e_ = 0.186). The obtained data are summarized in Table 2, and the corresponding CIE chromaticity diagrams are displayed in Fig. 8. It is evident that upon 349 nm excitation, the chromaticity point changes systematically from cyan (0.2235, 0.3106) to orange-red (0.5714, 0.3549), by adjusting the Eu^3+^ content from *x* = 0 to 0.2. Among these phosphors, Ba_4_BiTb_0.999_Eu_0.001_O(BO_3_)_4_ can emit three colors (blue, green, and red from Bi^3+^, Tb^3+^, and Eu^3+^, respectively) simultaneously, resulting in near-white light emitting from the single-phase host. It demonstrates the CIE coordinates of (0.3365, 0.2938) and a CCT of 5242 K, which are close to the standard white-light illumination [(0.3333, 0.3333) and CCT = 5454.12 K].^69^ When *λ*_ex_ = 377 nm, the chromaticity point can be tuned almost linearly from green (0.2785, 0.5058) to orange (0.4854, 0.4389) and then to red (0.6120, 0.3797) by controlling the concentration ratio of Tb^3+^/Eu^3+^. For an intuitionistic display, digital photographs of the selected phosphors under n-UV irradiation are also presented in Fig. 8, where the near-white emission for Ba_4_BiTb_0.999_Eu_0.001_O(BO_3_)_4_ (*λ*_ex_ = 349 nm) and a variation in the emission color from green to red for Ba_4_BiTb_1−*x*_Eu_*x*_O(BO_3_)_4_ (*λ*_ex_ = 377 nm) are visible. Based on the CIE diagrams, it is expected that when the Eu^3+^ doping amount is greater than 0.2, the luminescence color of Ba_4_BiTb_1−*x*_Eu_*x*_O(BO_3_)_4_ will change slightly from orange-red to red for *λ*_ex_ = 349 nm. In comparison, it will remain red for *λ*_ex_ = 377 nm, due to the high efficiency of Bi^3+^-to-Eu^3+^ and Tb^3+^-to-Eu^3+^ energy transfer. What's more, the quantum yields (QYs) of Ba_4_BiTb_0.999_Eu_0.001_O(BO_3_)_4_ were determined by the integration sphere method to be approximately 0.06% for *λ*_ex_ = 349 nm. The QYs of Ba_4_BiTb_1−*x*_Eu_*x*_O(BO_3_)_4_ (*x* = 0, 0.001, 0.002, 0.005, 0.01, 0.05, 0.1, 0.2) for *λ*_ex_ = 377 nm were 0.25%, 0.51%, 0.63%, 1.02%, 1.49%, 3.65%, 4.75%, and 7.06%, respectively. The QYs of Ba_4_BiTb_1−*x*_Eu_*x*_O(BO_3_)_4_ phosphors (*λ*_ex_ = 377 nm) increase with the increase of Eu^3+^ concentration from *x* = 0 to 0.2, which is attributed to the efficient Tb^3+^ → Eu^3+^ energy transfer. The low QY values may be because the sample was prepared by a simple high-temperature solid-state reaction, and we believe that the luminous efficiency can be enhanced by optimizing the experimental conditions in future research.

**Table 2:** CIE chromaticity coordinates and CCT values of Ba_4_BiTb_1−*x*_Eu_*x*_O(BO_3_)_4_ (0 ≤ *x* ≤ 0.2) phosphors under the excitation of 349 and 377 nm

Ba_4_BiTb_1−*x*_Eu_*x*_B_4_O_13_	CIE(*x*, *y*) at *λ*_ex_=		CCT(*K*) at *λ*_ex_=	
	349 nm	377 nm	349 nm	377 nm
*x* = 0	0.2235	0.2785	14 437	6761
	0.3106	0.5058		
*x* = 0.001	0.3365	0.4854	5242	2578
	0.2938	0.4389		
*x* = 0.002	0.3886	0.5248	3173	2064
	0.3154	0.4235		
*x* = 0.005	0.3829	0.5545	3009	1708
	0.2921	0.3947		
*x* = 0.01	0.4231	0.5759	2275	1644
	0.3131	0.3946		
*x* = 0.05	0.5476	0.6004	1642	1623
	0.3697	0.3889		
*x* = 0.1	0.5703	0.6079	1620	1636
	0.3718	0.3865		
*x* = 0.2	0.5714	0.6120	1652	1667
	0.3549	0.3797		

### 3.9. Thermal stability

**Fig. 9:**
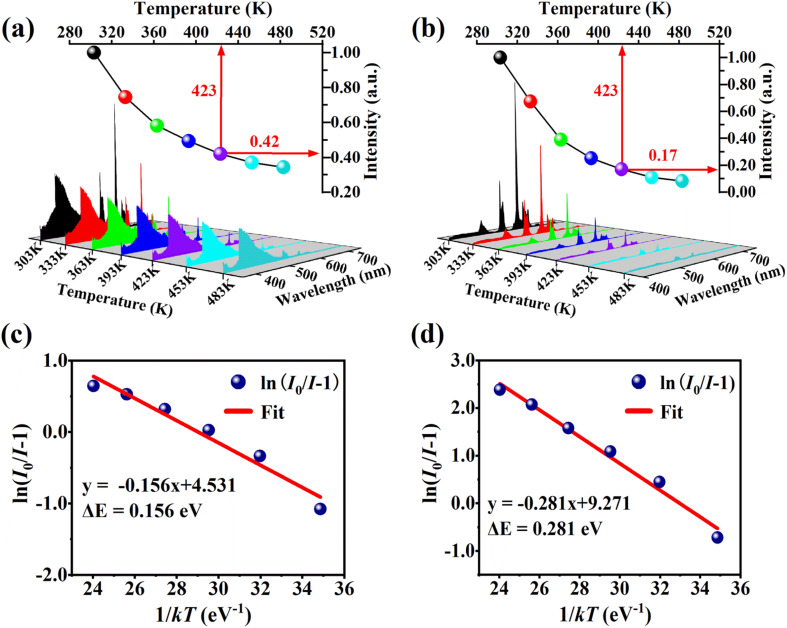
Temperature-dependent PL spectra of the Ba_4_BiTb_0.999_Eu_0.001_O(BO_3_)_4_ phosphor upon excitation at 349 nm (a) and 377 nm (b), respectively; the insets show the relative PL integral intensities of the phosphor. The linear fitting curves of ln(*I*_0_/*I* − 1) *versus* 1/*k*T for *λ*_ex_ = 349 nm (c) and *λ*_ex_ = 377 nm (d) are also depicted in the figure.

Thermal stability is an indispensable parameter for novel phosphor material because the LED chips are always operated in a high-temperature environment. The temperature-dependent PL spectra of the phosphor Ba_4_BiTb_0.999_Eu_0.001_O(BO_3_)_4_ were recorded under two excited wavelengths of 349 and 377 nm, respectively, and the corresponding normalized emission intensities were calculated, as shown in Fig. 9(a) and (b). The PL intensity decreases monotonically with the temperature increasing from 303 to 483 K due to the thermal quenching effect. When the temperature reaches 423 K (150 °C), the PL intensity retains ∼42% (*λ*_ex_ = 349 nm) and ∼17% (*λ*_ex_ = 377 nm) of that at room temperature (303 K), respectively, indicating that the thermal quenching is more obvious for *λ*_ex_ = 377 nm than *λ*_ex_ = 349 nm. To better understand the temperature dependence of photoluminescence, the activation energy of thermal quenching (Δ*E*) was calculated by the Arrhenius equation given as:^70^6
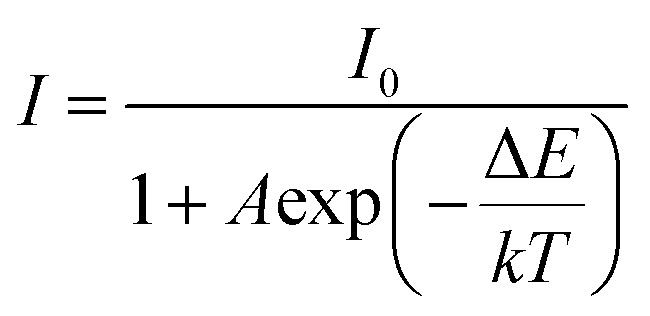
where *I* and *I*_0_ represent the PL intensity at temperatures of *T* and 303 K, respectively, and *A* and *k* are constants (*k* = 8.62 × 10^−5^ eV K^−1^). Fig. 9(c) and (d) show the plots of ln(*I*_0_/*I* − 1) against 1/*kT* for this phosphor under two excitations. The data can be well-fitted to straight lines with slopes of −0.156 and −0.281, respectively. Thus, the obtained Δ*E* value of 0.281 eV for *λ*_ex_ = 377 nm is greater than that of 0.156 eV for *λ*_ex_ = 349 nm, and both are comparable to those of several previously reported Bi^3+^/Tb^3+^/Eu^3+^ co-doped phosphors, such as Ca_2.24_ZrSi_2_O_9_:0.17Eu^3+^, 0.09Bi^3+^, 0.50Tb^3+^ (Δ*E* = 0.195 eV), Gd_1.474_MoB_2_O_9_:0.30Bi^3+^, 0.20Tb^3+^, 0.026Eu^3+^, (Δ*E* = 0.17 eV) and LaMoBO_6_:0.4Tb^3+^, 0.005Eu^3+^, 0.02Bi^3+^ (Δ*E* = 0.19 eV).^30,31,71^

**Fig. 10:**
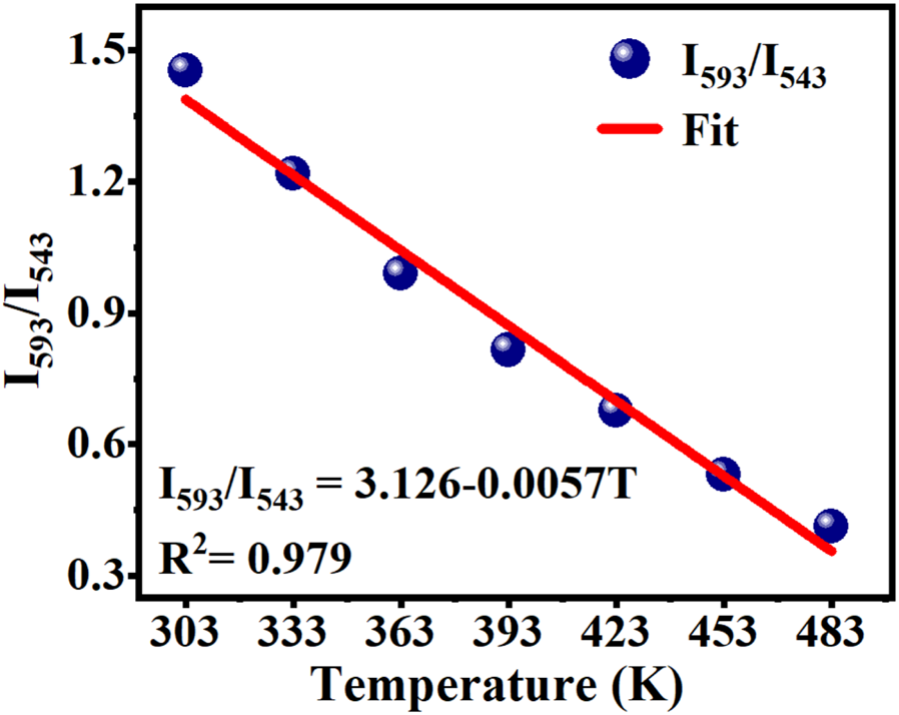
Temperature-dependent FIR (FIR = *I*_593_/*I*_543_, where *I*_593_ and *I*_543_ are the integrated emission intensities of Eu^3+^:^5^D_0_ → ^7^F_1_ transition from 585 to 600 nm and Tb^3+^:^5^D_4_ → ^7^F_5_ transition from 535 to 565 nm, respectively) of the Ba_4_BiTb_0.999_Eu_0.001_O(BO_3_)_4_ phosphor upon excitation at 377 nm. The fitted line for the experimental data is also provided in the figure.

This phosphor exhibits strong temperature sensitization when excited at 377 nm, and its CIE coordinates shift significantly from (0.4540, 0.4052) to (0.2891, 0.3308) as the temperature increases from 303 to 483 K, indicating its potential application in temperature sensing. Fig. 10 shows the temperature-dependent Eu^3+^/Tb^3+^ fluorescence intensity ratio FIR [FIR = *I*_593_/*I*_543_ = *I*(Eu^3+^:^5^D_0_ → ^7^F_1_)/*I*(Tb^3+^:^5^D_4_ → ^7^F_5_)] for this phosphor upon excitation at 377 nm. The calculated FIR-value monotonically decreases with rising temperature from 303 to 483 K, which can be fitted to the correlation coefficient *R*^2^ = 0.979 employing the linear function described by the equation:

7 FIR = *I*_593_/*I*_543_ = *A* + *BT* = 3.126 − 0.0057*T*

Thus, the sensitivity of Ba_4_BiTb_0.999_Eu_0.001_O(BO_3_)_4_ for temperature sensing is about 0.57% per K. Considering this change in the emission intensity and chromaticity coordinates with temperature, we expect that Ba_4_BiTb_1−*x*_Eu_*x*_O(BO_3_)_4_ phosphors excited at 377 nm may be used as luminescent thermometers at this temperature range.^72,73^ A detailed investigation of this aspect is beyond the scope of this article and will be provided elsewhere.

## 4. Conclusions

The investigation of the BaO (and SrO)–Bi_2_O_3_–Tb_2_O_3_–B_2_O_3_ system led to the discovery of two new phases, Ba_4_BiTbO(BO_3_)_4_ and Ba_1.54_Sr_2.46_BiTbO(BO_3_)_4_. They are layered compounds, where Bi_2_O_13_ groups share corners with each other and edges with BO_3_ triangles to create a 2D [Bi(BO_3_)_2_O]_*n*_^5*n*−^ layer. There exists another kind of 2D layer, [Tb(BO_3_)_2_]_*n*_^3*n*−^, constructed from TbO_6_ octahedra and BO_3_ triangles. These layers and their equivalent partners are stacked alternately along the [001] direction, with the interlayer spaces and intralayer tunnels filled by alkali-earth cations. This study also reveals that Ba_4_BiLnO(BO_3_)_4_ (Ln = trivalent rare-earth ions) forms a large family of compounds, in which Ba^2+^ can be partially replaced by other divalent cations, such as Pb^2+^ and Sr^2+^. Additionally, a series of phosphors, Ba_4_BiTb_1−*x*_Eu_*x*_O(BO_3_)_4_ (0 ≤ *x* ≤ 1) and Ba_4_BiYO(BO_3_)_4_, were synthesized at 830 °C. Based on analyses of IR/Raman and UV-vis diffuse reflection spectra, the geometric deviation of the BO_3_ group from the *D*_3h_ symmetry was verified, and the indirect and direct band gaps of Ba_4_BiLnO(BO_3_)_4_ (Ln = Y, Tb, Eu) were determined. Upon 349 nm excitation, the PL spectra of Ba_4_BiTb_1−*x*_Eu_*x*_O(BO_3_)_4_ contain blue Bi^3+^:^3^P_1_ → ^1^S_0_, green Tb^3+^:^5^D_4_ → ^7^F_6,5_ and orange Eu^3+^:^5^D_0_ → ^7^F_1,2,3,4_ emissions. With an increase in Eu^3+^ content (*x*), the emission intensities of Bi^3+^ and Tb^3+^ decline steadily, whereas that of Eu^3+^ initially increases until *x* = 0.05, and then decreases. When *λ*_ex_ = 377 nm, only Tb^3+^ and Eu^3+^ emissions appear simultaneously, and the emission intensity of Tb^3+^ decreases, while that of Eu^3+^ increases along with increasing *x*. Thus, a single-component near-white emission and a color-tunable emission from green to red can be realized in Ba_4_BiTb_1−*x*_Eu_*x*_O(BO_3_)_4_ by manipulating the Tb^3+^/Eu^3+^ ratio and adopting different excitation wavelengths. Moreover, the Eu^3+^/Tb^3+^ fluorescence intensity ratio (*I*_593_/*I*_543_) for Ba_4_BiTb_0.999_Eu_0.001_O(BO_3_)_4_ upon 377 nm excitation can be linearly related to the temperature between 303 and 483 K, indicating the potential of the phosphor as a promising temperature sensor. These findings indicate that exploring new borates based on element substitution is feasible. By doping rare-earth activators onto the newly prepared borate matrix, multifunctional fluorescent materials can be obtained, which may find potential use for w-LEDs and temperature sensor applications. This is a promising field of research that is worth further studying.

## Conflicts of interest

There are no conflicts to declare.

## Supplementary Material

RA-014-D3RA08265B-s001

RA-014-D3RA08265B-s002
