# Supplementary material for: Two new rare-earth oxyborates Ba4BiTbO(BO3)4 and Ba1.54Sr2.46BiTbO(BO3)4 and luminescence properties of the Ba4BiTb1−xEuxO(BO3)4 phosphors
Source: RSC Adv. 2024 Feb 19;14(9):6270–84. doi: 10.1039/d3ra08265b (PMC10875415; doi:10.1039/d3ra08265b)
Supplement: RA-014-D3RA08265B-s001 [file RA-014-D3RA08265B-s001.pdf]

Electronic Supplementary Material (ESI) for RSC Advances.
This journal is © The Royal Society of Chemistry 2024

Supporting Information

Two new rare-earth oxyborates $Ba_4BiTbO(BO_3)_4$ and $Ba_{1.54}Sr_{2.46}BiTbO(BO_3)_4$ and luminescent properties of the $Ba_4BiTb_{1-x}Eu_xO(BO_3)_4$ phosphors

Xuean Chen[a,*], Xuyang Yuan[a], Weiqiang Xiao[b] and Xiaoyan Song[a]

[a]*Faculty of Materials and Manufacturing, Key Laboratory of Advanced Functional Materials, Ministry of Education of China, Beijing University of Technology, 100124 Beijing, China.*

E-mail:xueanchen@bjut.edu.cn

[b]*Beijing Key Laboratory of Microstructure and Property of Solids, Beijing University of Technology, 100124 Beijing, China*

Content

1. **Table S1** Atomic coordinates, site occupancies and equivalent isotropic displacement parameters (Å^2) for $Ba_4BiTbO(BO_3)_4$ and $Ba_{1.54}Sr_{2.46}BiTbO(BO_3)_4$.
2. **Table S2** Selected bond lengths (Å) and angles (°) for $Ba_4BiTbO(BO_3)_4$.
3. **Table S3** Selected bond lengths (Å) and angles (°) for $Ba_{1.54}Sr_{2.46}BiTbO(BO_3)_4$.
4. **Table S4** Rietveld refinement results of $Ba_4BiTb_{1-x}Eu_xO(BO_3)_4$ ($0 \le x \le 1$) and $Ba_4BiYO(BO_3)_4$.
5. **Fig. S1** A FE-SEM image, EDX results and elemental mapping of a typical $Ba_4BiTbO(BO_3)_4$ single crystal.
6. **Fig. S2** A FE-SEM image, EDX results and elemental mapping of a typical $Ba_{1.54}Sr_{2.46}BiTbO(BO_3)_4$ single crystal.
7. **Fig. S3** Rietveld plots of $Ba_4BiTb_{1-x}Eu_xO(BO_3)_4$ (x = 0.001, 0.002, 0.005, 0.01, 0.05, 0.1, and 0.2) and $Ba_4BiYO(BO_3)_4$.
8. **Fig. S4** Infrared and Raman spectra of $Ba_4BiLnO(BO_3)_4$ (Ln = Y, Tb and Eu).
9. **Fig. S5** XPS survey (a) and core-level spectra of Ba 3d (b), Bi 4f (c), Tb 3d (d), Eu 3d (e), B 1s (f) and O 1s (g) for $Ba_4BiTb_{0.999}Eu_{0.001}O(BO_3)_4$.
10. **Fig. S6** UV–vis diffuse reflection spectra of $Ba_4BiLnO(BO_3)_4$ (Ln = Y, Tb and Eu) (left). Tauc plots for indirect (middle) and direct (right) transitions are also shown in this figure.

Table S1 Atomic coordinates, site occupancies and equivalent isotropic displacement parameters (Å^2) for $Ba_4BiTbO(BO_3)_4$ and $Ba_{1.54}Sr_{2.46}BiTbO(BO_3)_4$.

Atoms	Wyck sites	Site symmetry	x	y	z	Occupancies	U_{eq}
$Ba_4BiTbO(BO_3)_4$							
Ba1	4*f*	C_{3v}	0.6667	0.3333	0.08794(3)	1	0.0146(2)
Ba2	2*d*	D_{3h}	0.6667	0.3333	0.2500	1	0.0160(2)
Ba3	4*e*	C_{3v}	0.0000	0.0000	0.15797(9)	0.5	0.0132(4)
Bi1	4*e*	C_{3v}	0.0000	0.0000	0.17011(6)	0.5	0.0355(6)
Tb1	2*a*	D_{3d}	0.0000	0.0000	0.0000	1	0.00881(19)
B1	4*f*	C_{3v}	0.3333	0.6667	0.0538(6)	1	0.011(2)
B2	4*f*	C_{3v}	0.3333	0.6667	0.1702(5)	1	0.012(2)
O1	12*k*	C_s	0.1871(6)	0.3742(12)	0.0537(2)	1	0.0204(11)
O2	12*k*	C_s	0.0396(14)	0.5198(7)	0.16991(19)	1	0.0218(12)
O3	2b	D_{3h}	0.0000	0.0000	0.2500	1	0.029(3)
$Ba_{1.54}Sr_{2.46}BiTbO(BO_3)_4$							
Ba1/Sr1	4*f*	C_{3v}	0.6667	0.3333	0.08892(5)	0.58(2)/0.42(2)	0.0163(5)
Ba2/Sr2	2*d*	D_{3h}	0.6667	0.3333	0.2500	0.38(3)/0.62(3)	0.0170(7)
Sr3	4*e*	C_{3v}	0.0000	0.0000	0.15810(19)	0.5	0.0062(7)
Bi1	4*e*	C_{3v}	0.0000	0.0000	0.17066(9)	0.5	0.0307(7)
Tb1	2*a*	D_{3d}	0.0000	0.0000	0.0000	1	0.0199(4)
B1	4*f*	C_{3v}	0.3333	0.6667	0.0548(11)	1	0.020(4)
B2	4*f*	C_{3v}	0.3333	0.6667	0.1725(10)	1	0.017(3)
O1	12*k*	C_s	0.1857(12)	0.371(2)	0.0554(4)	1	0.032(2)
O2	12*k*	C_s	0.035(2)	0.5177(11)	0.1721(4)	1	0.026(2)
O3	2*b*	D_{3h}	0.0000	0.0000	0.2500	1	0.029(4)

Note: U_{eq} is defined as one third of the trace of the orthogonalized **U** tensor.

Table S2 Selected bond lengths (Å) and angles (°) for $Ba_4BiTbO(BO_3)_4$.

Ba1-O2 × 3	2.780(6)	Bi1-O3	2.1055(15)
Ba1-O1 × 6	2.862(2)	Bi1-O2 × 6	2.7157(5)
Ba2-O2 × 6	2.742(6)	Tb1-O1 × 6	2.255(6)
Ba2-O3 × 3	3.12846(5)	B1-O1 × 3	1.372(6)
Ba3-O3	2.425(2)	B2-O2 × 3	1.379(7)
Ba3-O2 × 6	2.7339(8)	Ba3-Bi1	0.320(3)
O1-Tb1-O1 × 3	180.0(5)	O1-B1-O1 × 3	119.999(14)
O1-Tb1-O1 × 6	84.8(2)	O2-B2-O2 × 3	119.998(15)
O1-Tb1-O1 × 6	95.2(2)		

Table S3 Selected bond lengths (Å) and angles (°) for $Ba_{1.54}Sr_{2.46}BiTbO(BO_3)_4$.

Ba1/Sr1-O2 × 3	2.763(9)	Bi1-O3	2.068(2)
Ba1/Sr1-O1 × 6	2.827(4)	Bi1-O2 × 6	2.6880(6)
Ba2/Sr2-O2 × 6	2.657(10)	Tb1-O1 × 6	2.250(11)
Ba2/Sr2-O3 × 3	3.09768(11)	B1-O1 × 3	1.372(11)
Sr3-O3	2.396(5)	B2-O2 × 3	1.384(10)
Sr3-O2 × 6	2.7123(16)	Sr3-Bi1	0.328(5)
O1-Tb1-O1 × 3	180.0(5)	O1-B1-O1 × 3	119.99(6)
O1-Tb1-O1 × 6	83.3(4)	O2-B2-O2 × 3	119.99(4)
O1-Tb1-O1 × 6	96.7(4)		

Table S4 Rietveld refinement results of $Ba_4BiTb_{1-x}Eu_xO(BO_3)_4$ ($0 \le x \le 1$) and $Ba_4BiYO(BO_3)_4$.

**Eu^{3+} content**	x=0	x=0.001	x=0.002	x=0.005	x=0.01
**Space group**	$P6_3/mmc$	$P6_3/mmc$	$P6_3/mmc$	$P6_3/mmc$	$P6_3/mmc$
***a* (Å)**	5.42269(15)	5.4231(3)	5.4236(5)	5.4238(4)	5.4243(4)
***c* (Å)**	26.3902(9)	26.4080(14)	26.413(2)	26.411(2)	26.409(2)
***V* (Å³)**	672.05(4)	672.60(8)	672.83(13)	672.86(12)	672.93(11)
**2*θ* range (°)**	10–90	10–90	10–90	10–90	10–90
**R_p, %**	4.17	4.31	3.95	3.90	4.00
**R_{wp}, %**	7.89	7.86	7.17	7.10	7.30
**GOF**	4.77	3.55	3.15	3.11	3.21

**Eu^{3+} content**	x=0.05	x =0.1	x =0.2	x =1	$Ba_4BiYO(BO_3)_4$
**Space group**	$P6_3/mmc$	$P6_3/mmc$	$P6_3/mmc$	$P6_3/mmc$	$P6_3/mmc$
***a* (Å)**	5.4245(4)	5.4256(5)	5.4257(4)	5.4322(3)	5.4160(9)
***c* (Å)**	26.410(2)	26.416(2)	26.423(2)	26.5305(16)	26.289(4)
***V* (Å³)**	673.02(12)	673.43(13)	673.62(11)	677.99(8)	667.8(2)
**2*θ* range (°)**	10–90	10–90	10–90	10–90	10–90
**R_p, %**	3.88	4.13	4.20	5.64	5.97
**R_{wp}, %**	6.98	7.42	7.63	10.33	10.73
**GOF**	3.04	3.31	3.41	6.69	6.52

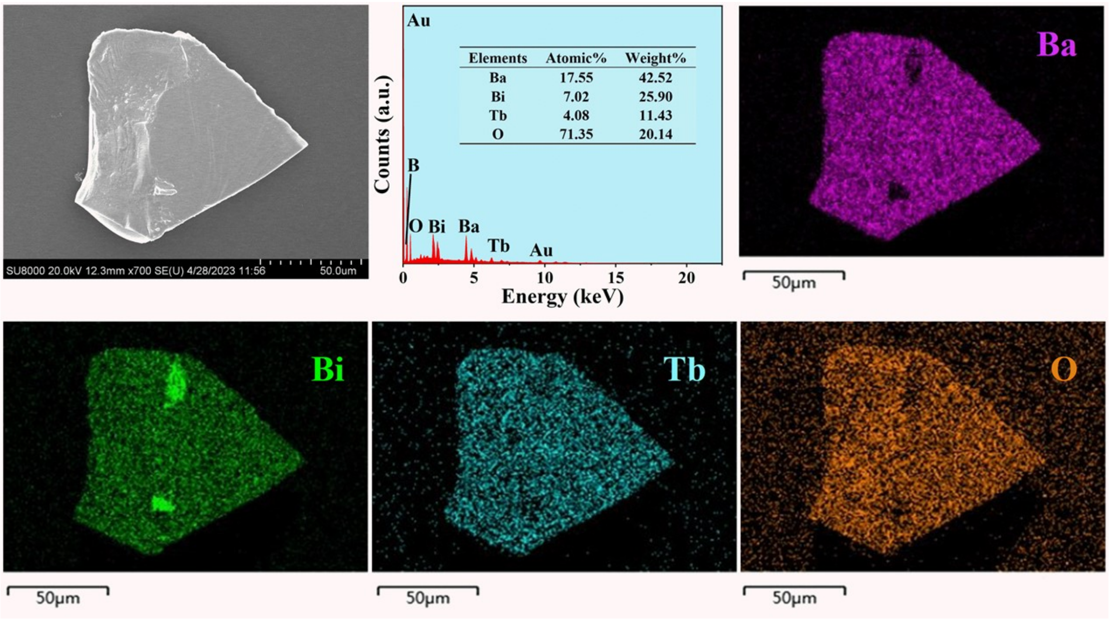

Fig. S1 A FE-SEM image, EDX results and elemental mapping of a typical $Ba_4BiTbO(BO_3)_4$ single crystal. The Au element comes from the pretreatment process (A thin layer of Au was evaporated on the sample surface to provide electrical conductivity).

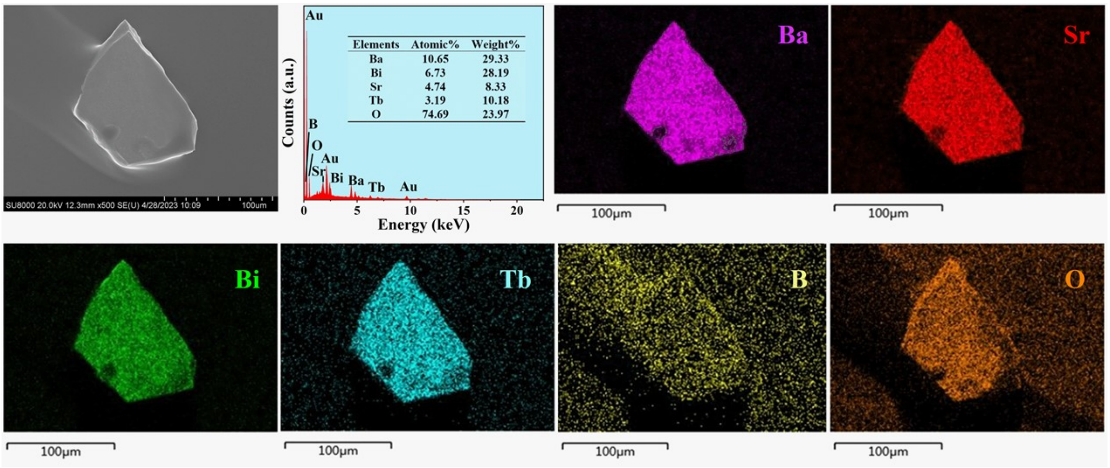

Fig. S2 A FE-SEM image, EDX results and elemental mapping of a typical $Ba_{1.54}Sr_{2.46}BiTbO(BO_3)_4$ single crystal.

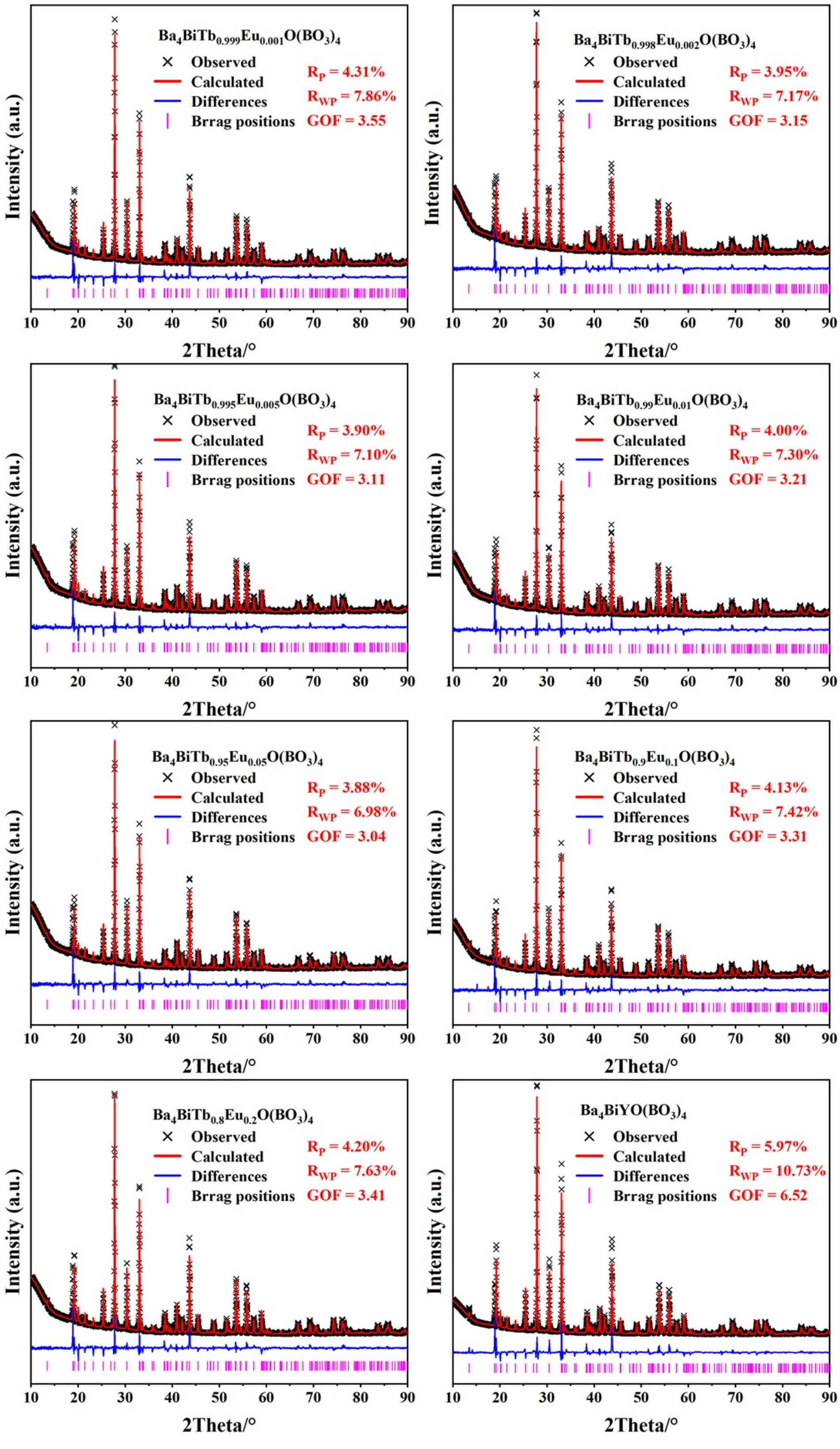

Fig. S3 Rietveld plots of $Ba_4BiTb_{1-x}Eu_xO(BO_3)_4$ (x = 0.001, 0.002, 0.005, 0.01, 0.05, 0.1, and 0.2) and $Ba_4BiYO(BO_3)_4$.

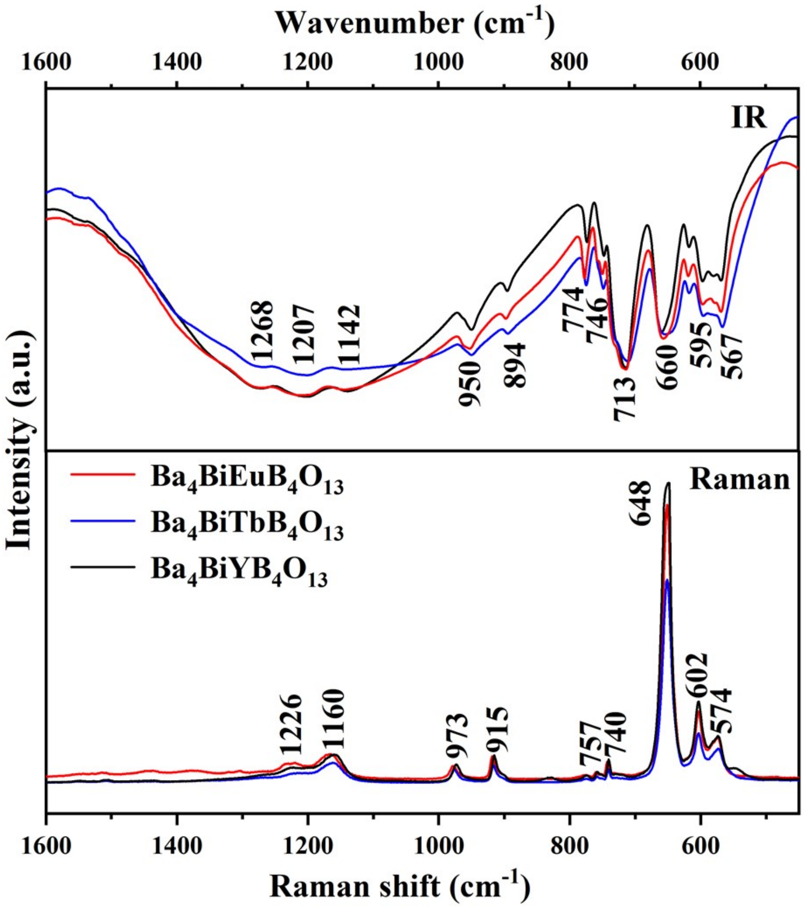

Fig. S4 Infrared and Raman spectra of $Ba_4BiLnO(BO_3)_4$ (Ln = Y, Tb and Eu).

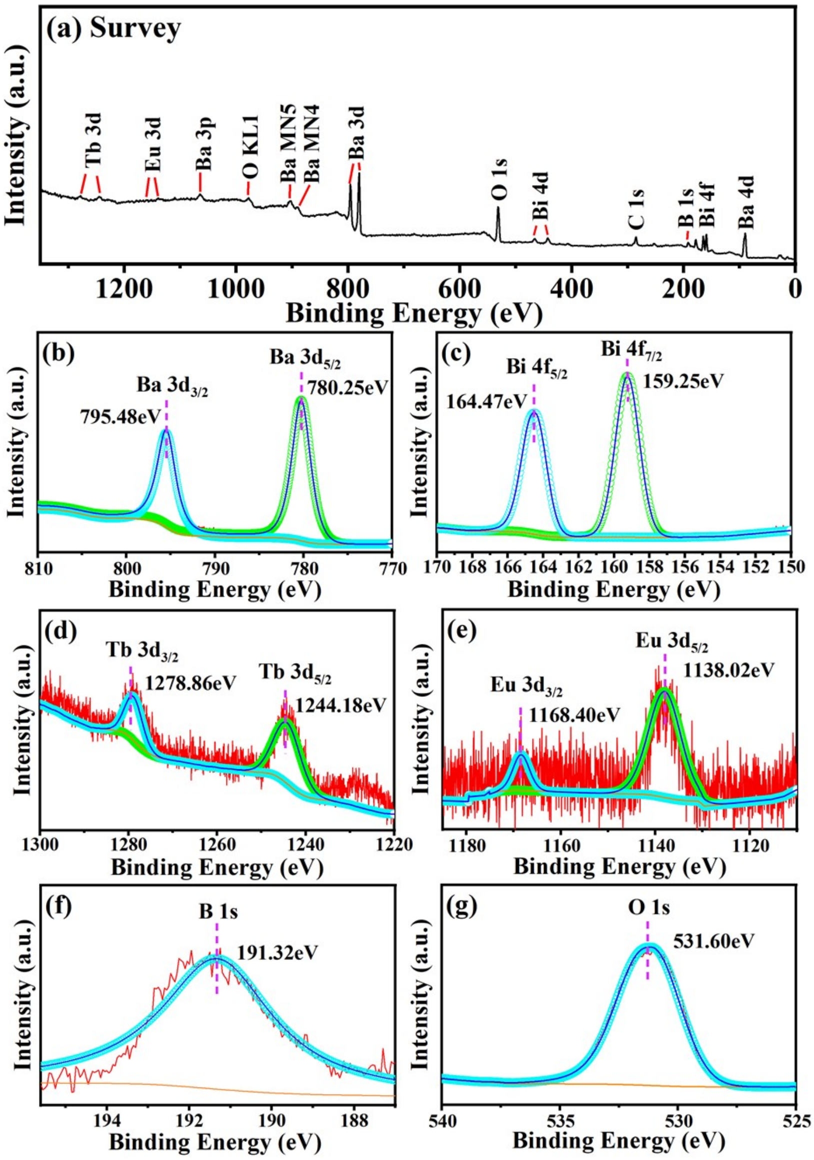

Fig. S5 XPS survey (a) and core-level spectra of Ba 3d (b), Bi 4f (c), Tb 3d (d), Eu 3d (e), B 1s (f) and O 1s (g) for $Ba_4BiTb_{0.999}Eu_{0.001}O(BO_3)_4$.

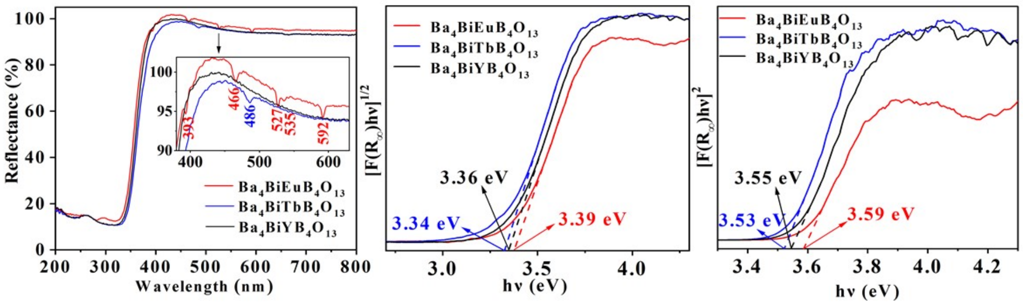

Fig. S6 UV–vis diffuse reflection spectra of $Ba_4BiLnO(BO_3)_4$ (Ln = Y, Tb and Eu) (left). Tauc plots for indirect (middle) and direct (right) transitions are also shown in this figure.
